# Hydrogel-Based Biosensors

**DOI:** 10.3390/gels8120768

**Published:** 2022-11-25

**Authors:** Katharina Völlmecke, Rowshon Afroz, Sascha Bierbach, Lee Josephine Brenker, Sebastian Frücht, Alexandra Glass, Ryland Giebelhaus, Axel Hoppe, Karen Kanemaru, Michal Lazarek, Lukas Rabbe, Longfei Song, Andrea Velasco Suarez, Shuang Wu, Michael Serpe, Dirk Kuckling

**Affiliations:** 1 Department of Chemistry, Universität Paderborn, Warburger Straße 100, 33098 Paderborn, Germany; 2 Department of Chemistry, University of Alberta, 11227 Saskatchewan Drive, Edmonton, AB T6G 2G2, Canada

**Keywords:** hydrogel, biosensor, biomarker, protein, DNA, drug, environmental pollutant

## Abstract

There is an increasing interest in sensing applications for a variety of analytes in aqueous environments, as conventional methods do not work reliably under humid conditions or they require complex equipment with experienced operators. Hydrogel sensors are easy to fabricate, are incredibly sensitive, and have broad dynamic ranges. Experiments on their robustness, reliability, and reusability have indicated the possible long-term applications of these systems in a variety of fields, including disease diagnosis, detection of pharmaceuticals, and in environmental testing. It is possible to produce hydrogels, which, upon sensing a specific analyte, can adsorb it onto their 3D-structure and can therefore be used to remove them from a given environment. High specificity can be obtained by using molecularly imprinted polymers. Typical detection principles involve optical methods including fluorescence and chemiluminescence, and volume changes in colloidal photonic crystals, as well as electrochemical methods. Here, we explore the current research utilizing hydrogel-based sensors in three main areas: (1) biomedical applications, (2) for detecting and quantifying pharmaceuticals of interest, and (3) detecting and quantifying environmental contaminants in aqueous environments.

## 1. Introduction

Hydrogels provide a tremendous opportunity to serve as sensory systems as they are oftentimes biocompatible and resemble hydrated biological tissues. Therefore, they are potentially applicable in vivo. Furthermore, their synthetic variability allows for fine tuning of the gel chemistry to yield responses to desired stimuli [1]. These responses allow for direct (visual colour changes) and/or indirect (changes in UV absorption properties) read-outs through swelling/deswelling and degradation [2]. However, the characteristics and potential applications of hydrogels depends on both the preparation methods and the monomer used for hydrogel synthesis [3].

While hydrogels offer tremendous diversity in responsivity to a broad spectrum of stimuli, using them for sensing applications requires a signal transduction mechanism that will convert the hydrogel response into a readable signal. In one early example, hydrogels coloured with embedded nano/microparticles changed colour in the presence of glucose [4]. Electrochemical-based biosensors have proven to be useful in a wide range of applications and have become the most commonly used class of biosensors [5]. Their construction, consisting of a biological sensing element, a transducer, and an electronic system, allows for the fast determination of analyte concentration over a wide linear range [5,6,7]. Biosensors can be classified based on the recognition process utilized to detect the target analytes. Four main recognition processes are employed; (i) affinity sensors, which are based on specific binding interactions; (ii) conductometric sensors, which measure the change in conductance when the sensor is exposed to the analyte for quantification; (iii) potentiometric sensors, which measure the change in potential across a working electrode compared to a reference electrode; and (iv) amperometric biosensors, which measure the change in current resulting from the sensor interacting with the analyte [5,7]. Hydrogels have proven useful in electrochemical-based biosensors, serving as an immobilization matrix for the biosensing elements. Their three-dimensional structure provides an enhanced surface area, increasing the number of recognition elements. Additionally, hydrogels perform well under physiological conditions, allowing them to detect biological phenomena in vivo with minimal impact on the biological activity. Techniques and approaches have been developed to passivate the sensor surface to minimize undesired interactions in cases of in vivo sensing [8,9].

This review will focus on highlighting the utility of hydrogels for the detection of various classes of analytes utilizing a multitude of transduction mechanisms. Specifically, we focus on the applications for the detection of (1) important biomolecules pertaining to biomedical applications, including enzymes, hormones, metabolites, and DNA, (2) pharmaceutical and illicit drugs, and (3) common environmental pollutants, including heavy metal ions and pesticides.

## 2. Biomedical Applications

Biomarkers are a measurable entity that is indicative of a certain state of a living system [10]. Biomarker analysis is often concerned with the specific concentration of the biomarker in a sample, with concentrations outside a normal range indicating the presence of the disease [10]. Additionally, biomarkers are often molecular or biomolecular in nature, although there are biomarkers that are not molecular in nature, such as circulating tumor cells [11]. Specific biomolecules are produced in response to changes in environment, including stress and disease. These specific biomolecules can be detected and quantified to classify these changes in physiology [12]. Biomarker detection can facilitate the early detection of diseases before they enter the acute period, allowing for early intervention with less invasive procedures. Efficacy of treatments can be monitored in “real time” to track treatment progress. The study of biomarkers and assigning them to specific diseases is a rapidly growing field, with the number of publications increasing exponentially since 2005 [13]. Currently, thousands of chemical species have been identified as reliable biomarkers for a wide range of diseases, ranging from monatomic ions [14], small molecular markers [15,16], and even macromolecular biomarkers such as proteins or microRNA [17]. Many different systems have been developed and employed for the in vitro and in vivo detection and quantification of specific biomarkers, ranging from electrochemical assays and electrophoresis to spectroscopic methods such as fluorescence and Raman spectroscopy [18].

### 2.1. Proteins and Enzymes

Proteins play a vital role in the biochemistry of cells in every living organism. As enzymes, they serve as catalysts to allow highly specific and selective reactions. While the concentration of different enzymes in each organism naturally varies over time, an over- or under-expression of specific proteins can serve as a biomarker for a variety of diseases. One such group of enzymes are proteases which, when overexpression occurs, can be an indicator for cardiovascular and inflammatory diseases, or different types of cancer [19]. West and Hubbell pioneered research in this area by creating the first protease-responsive hydrogels in 1999 [20]. The hydrogels were prepared through photoinitiation of poly(ethyleneglycol) (PEG) diacrylate monomers that contained one of two different trimeric and tetrameric amino acid sequences, VGN and APGL, cleavable by the protease’s matrix metalloproteinase-1 (MMP-1) and plasmin, respectively. Upon exposure to solutions of the specific enzymes, the swollen hydrogels displayed complete and selective degradation after 5 days (APGL; 2 mg/mL) and 17 days (VRN; 2 U/mL) through proteolysis of the specific amino acid sequence, while no cleavage was observed when exposed to the other enzyme. Based on these results, a number of MMP-responsive hydrogels have since been studied as potential protein-responsive drug delivery systems [19], while Purcell et al. had already performed the first proof of concept study of such systems in 2014 [21].

Hydrogels can also serve to detect the presence of specific enzymes and release drugs in vitro. Kono et al. prepared an enzyme-targeted polysaccharide hydrogel, designed to release drugs when exposed to sugar-degrading enzymes [22]. Their polysaccharide hydrogel was designed to be targeted by β-mannosidase, which would break the gel down and release protein-based drugs in the process. According to Kono et al., hydrogel was effective at delivering the model drugs, bovine serum albumin and lysozyme when exposed to β-mannosidase in vivo, with them degrading by 30–65% in the presence of β-mannosidase [22]. This strategy can be employed to develop highly targeted drug delivery platforms. For instance, this hydrogel-based approach could deliver a specific enzyme therapy to the small intestine, and the hydrogel would protect the sensitive enzyme from degradation in the stomach and only release the therapeutic enzyme when the hydrogel is exposed to enzymes only present in the small intestine. While the hydrogel developed by Kono et al. does not induce an electronic signal that allows for the quantification of β-mannosidase, it is still a biosensor as it elicits a response, the release of proteins, in response to the presence of β-mannosidase.

Cancer is a leading cause of death worldwide. The measurement of cancer biomarkers in tissue, serum, blood, and urine provides the necessary information for clinical cancer screening and disease diagnosis. Early screening and diagnosis allows for early intervention, increasing the survival rate of cancer patients [23]. Shohatee et al. developed poly(ethylene glycol diacrylate) (PEGDA) hydrogel nanoparticles for a suspension array that can be used for the quantification of vascular endothelial growth factor (VEGF). Elevated VEGF levels are signature biomarkers of aggressive (MDA-MB-231) and non-aggressive (MCF-7) breast cancer cells [24].

Microparticles were prepared by photocrosslinking of an antibody-incubated pre-polymer solution using a mask. The microparticles were collected and incubated with streptavidin-modified horseradish peroxidase (HRP) that induced the reduction (and precipitation) of silver upon addition of a silver salt solution. In this step, H_2_O_2_ is generated in a redox reaction that at the same time reduces Ag^+^ to Ag^0^ which were deposited within the hydrogel matrices. In microparticles with protein association, a discolouration was observed, while negative samples showed a lower relative intensity. The silver precipitation readout was carried out through optical microscopy, permitting the quantitation of VEGF in a sample [24]. The resistance of PEGDA to protein adsorption reduces non-specific protein binding and thereby improves the signal-to-noise ratio by decreasing the background signal. The test allows analysis in under one hour, with a detection limit of 5.2 ± 1.0 pg/mL. This detection limit corresponds to healthy patient VEGF levels and exceeds the performance of conventional methods, which allows for broad monitoring.

**Figure 1:**
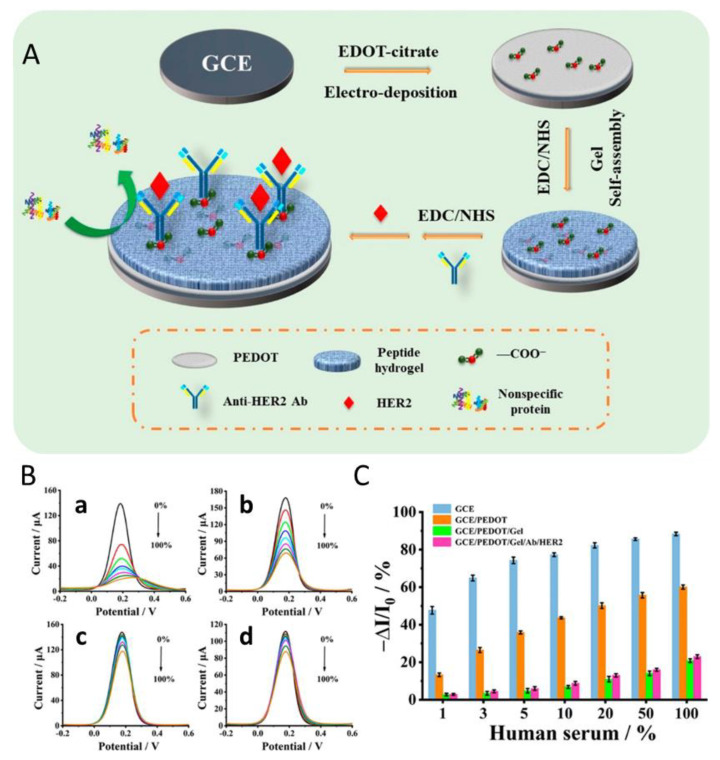
(**A**) Schematic representation of the PEDOT/peptide hydrogel sensor preparation. (**B**) DPV responses of (**a**) bare GCE, (**b**) GCE/PEDOT, (**c**) GCE/PEDOT/gel. and (**d**) GCE/PEDOT/Gel/Ab/HER2 after incubation in human sera with different concentrations. (**C**) The corresponding signal change rates of different electrodes. Error bars show the standard deviations of three repeated measurements (Reprinted and adapted with permission from [25]. Copyright 2022 American Chemical Society).

In another study, Wang et al. used hydrogels for the electrochemical detection of the breast cancer biomarker HER2 [25]. They developed a biosensor with an antifouling sensing interface based on the highly stable conducting polymer poly (3,4-ethylenedioxythiophene) and a biocompatible peptide hydrogel (FEKF functionalized with a fluorene methoxycarbonyl group) that provided effective alleviation of nonspecific adsorption or biofouling (Figure 1). For the detection of targets, HER2 samples of different concentrations were prepared in phosphate-buffered saline. The reduction of the biosensor signal was recorded by differential pulse voltammetry (DPV) that indicates the amount of the biomarker present in the serum sample. This developed biosensor was selective for HER2 in human serum with a linear response range from 0.1 ng/mL to 1.0 μg/mL and a lower limit of detection (LOD) of 45 pg/mL. Not only does the good sensitivity of this hydrogel sensor make it suitable for medical applications, but it also demonstrates that this test exhibits clinically acceptable accuracy in complex biological media (Figure 1) [25].

The measurement of protein biomarkers not only allows for the detection of different diseases, but also aids in monitoring treatment efficacy. For these types of measurements, enzyme-linked immunosorbent assays (ELISAs) are widely employed due to the breadth of analytes capable of being detected and the high throughput [26]. The typical ELISA method has some drawbacks as it requires highly skilled technicians, expensive instrumentation, demands expert skills for diagnosis, and is time consuming. Various research has been carried out to develop one-step plasmonic ELISA where metal nanoparticles that exhibit particular colours based on localized surface plasmon resonance (LSPR) (e.g., gold, copper, silver) are being used [26]. Moreover, electrochemical immunosensors are widely used due to their high sensitivity, efficiency, low cost, and user-friendly instrumentation, especially for label-free electrochemical immunoassays [27]. Wang et al. (2017) fabricated a label-free electrochemical immunosensor to detect the lung cancer biomarker neuron-specific enolase (NSE) in human serum samples [28]. This immunosensor utilized a conductive hydrogel, synthesized through the cross-linking coordination technique from a mixture of 1,3,5-benzenetricarboxylic acid and Fe^3+^-ions. A thin homogeneous film of the hydrogel was made on a glassy carbon electrode by a drop coating method followed by the immobilization of antibodies on the surface. To increase the conductivity and immobilization, gold nanoparticles (AuNPs) were uniformly deposited onto this film, which resulted in an amperometric NSE sensor. The signal read-out was through square wave voltammetry (SWV) and electrochemical impedance spectroscopy (EIS). The sensor showed a broad linear range (LDR) of 1 pg/mL to 200 ng/mL, and a low limit of detection of 0.26 pg/mL. This method’s accuracy was also comparable to ELISA-based assays.

The Ma group performed ultrasensitive amperometric detection of the tumor biomarker Cytokeratin antigen 21-1 (CYFRA21-1) in human serum. This label-free electrochemical immunosensor was prepared from the mixture of phytic acid and lead nitrate. Analogous to the preparation method as described above [29], the electrode was coated with the hydrogel film via drop coating from phytic acid and lead nitrate. Again, AuNPs were used to immobilize anti-NSE and anti-CYFRA21-1. For a label-free immunosensor, hydrogel plays a crucial role in its analytical performance. The metal ions in the hydrogel possess good conductivity and exhibit a strong electrochemical signal while the acid acts as a multi-complexing agent for them. This strategy for the fabrication of conductive hydrogels can be easily extended to other hydrogels with metal ions including Zn^2+^, Fe^3+^, and Cd^2+^. The electrochemical signal of this immunosensor depends on the concentration of the specific biomarker in the sample and it shows an increased LDR as compared to the previous sensor (50 fg/mL to 100 ng/mL) [28,29].

**Figure 2:**
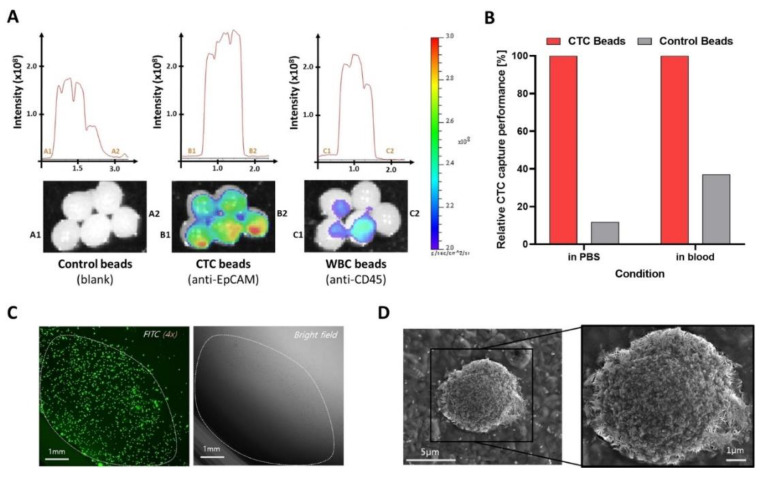
Performance of liquid biopsy hydrogel beads (LBBeads) for circulating tumor cell (CTC) recovery. (**A**) CTC capture performance comparison based on fluorescence intensities using fluorescent cancer cells with three different hydrogel beads; (**B**) CTC recovery performance after capture and release of cancer cells from the beads (**C**,**D**) Isolated fluorescent MCF-7 cancer cells under fluorescence microscope (**C**) and scanning electron microscope (SEM) (**D**) (Reprinted with permission from [31]. Copyright 2021 Wiley).

Liquid biopsy of circulating biomarkers in the blood stream is a technological revolution in medical science for the early diagnosis of several diseases, especially cancer. Unlike the conventional biopsy method which employs histology on solid tissue samples collected through invasive surgeries, liquid biopsy methods use bodily fluids including blood and urine for diagnosis [30]. Many researchers have been developing different techniques for the isolation and detection of circulating biomarkers. In their 2021 study, Kang et al. isolated circulating tumor cells (CTCs) and exosomes from a liquid biopsy [31]. They generated chemically stable and biodegradable hydrogel beads made of alginate and poly(vinylalcohol), which are advantageous over single component hydrogels, as they can endure multi-step processing and prolonged incubation periods. The bead surface was modified with anti-EpCAM and anti-CD63 antibodies and the finished particles were incubated with the respective circulating biomarkers, as well as control media. To detect positive results, fluorescent secondary antibodies were applied that showed distinctive patterns when analyzed using fluorescence microscopy (Figure 2). More interestingly, exosome-specific beads were prepared analogously to extract exosomes expressed by MCF-7 cancer cells from spiked samples.

Yamamoto et al. presented plasmonic ELISA to detect and measure human immunoglobulin G (hIgG) with a four-component detector system that consisted of poly(vinyl pyrrolidone) (PVP) hydrogels with silver nanoparticles (AgNPs) incorporated, a glucose oxidase (GOx) antibody release pad, a glucose release pad, and an antibody-immobilized membrane [26]. Unlike in other plasmonic ELISA methods that detect the change of the refractive index, the AgNPs were used as a chromogenic substrate to detect an absorbance peak intensity shift. This makes this method simple and an inexpensive optical system over the conventional ELISA method. This colour shift is induced by enzymatically-released H_2_O_2_. It is suggested that if positive samples were added to this system, released GOx antibodies would undergo an immunoreaction with the antigens and absorb into the antibody-immobilized membrane. Negative samples would pass through the membrane and release glucose that is enzymatically metabolized. Finally, the released H_2_O_2_ would reach the hydrogel in which the AgNPs were oxidized, resulting in a colour change from yellow to colourless. Positive samples would remain unchanged. The isolated components of this device were tested thoroughly; however, this device remains to be tested on hIgG samples.

Dry eye is a highly prevalent, yet often undiagnosed condition that causes irritation of the eye as well as more serious conditions that may require surgical procedures [32,33]. Dry eye and the accompanying irritation has become even more prevalent due to increased reading and screen time during the COVID-19 pandemic [34]. Tears are an easily accessible biological fluid for the assessment of protein biomarkers, making them an ideal fluid for liquid biopsies. ELISA is the most commonly used method for the determination of proteins in tears [35]. However, biosensors based on polymeric micro- and nanogels have allowed for new methods for dry eye diagnosis to be developed [35,36]. Among others, tears contain the proteins lysozyme, lactoferrin, IgA, and lipocalin-1, of which the first two show a high isoelectric point in comparison to the latter. Low concentrations of these proteins are a biomarker for dry eye conditions. Therefore, the simple detection of these proteins is not sufficient, but quantification is required for effective clinical application. For this reason, a label-free, LSPR-based method was developed by the Peppas group is an effective solution [36,37]. The hydrogel-coated gold nanoshells (AuNS) detected lysozyme and lactoferrin without interference of the low isoelectric point proteins IgA and lipocalin-1 [36]. In a previous study, the functionality of this system was explained by the formation of dynamic covalent imine-lysine bonds between the co-monomer oligo(ethylene glycol)acrylate (Al-OEGA) and the tear proteins [37]. Therefore the inclusion Al-OEGA into the poly(N-isopropylacrylamide-co-methacrylic acid) (p(NIPAM-co-MAA)) copolymers was studied in more detail [36]. A 2:1 ratio of MAA to Al-OEGA was found to be optimal, due to electrostatic interactions and led to significantly red-shifted LSRP signals in comparison to p(NIPAM-co-MAA)-coated AuNSs. Colourimetric detection was performed with a microplate plate reader, which allowed the automation of this process. This biosensor has potential applications as an affordable screening method for both chronic dry eye and the related diseases. In a clinical environment, these hydrogel-based biosensors facilitate affordable, rapid, and easy application for medical diagnostics compared to traditional in vitro diagnostic tests [36,37].

Acute myocardial infarction (AMI), or heart attack, is one of the leading causes of death throughout the world and is responsible for the occurrence of critical life-threatening conditions. The clinical confirmation of AMI and heart failure is performed through the detection of cardiovascular biomarkers such as cardiac troponin I (cTnI), B-type natriuretic peptide (BNP), and myoglobin (Myo) [38]. Ji et al. presented a biosensor composed of porous hydrogel-encapsulated photonic crystals (PhCs) to detect and quantify these biomarker proteins [39]. PhCs are challenging to employ in biosensors due to slow diffusion kinetics. Nevertheless, this barcode-based suspension array allowed for the fast and simultaneous detection of multiple biomarkers from a serum sample because of the hydrogel microcarriers. The hydrogel was made of PEGDA, which is non-toxic and ensured the stability of the core-shell structure, and gelatin that promoted target binding. The fluorescence intensities obtained from samples containing antigens were detected with fluorescence microscopy. The PhC crystals exhibited a stable diffraction peak for the corresponding markers and could differentiate probe-specific targets. Probes binding to the target caused the shells to shrink, which was decoded as a corresponding blue shift that was used for quantifying the amount of the biomarker or target. Moreover, because of the high surface to volume ratio of this biosensor, more fluorescent probe could be loaded in the particle, which helped to increase the sensitivity. The linear detection ranges were 0.01 to 1000 ng/mL for cTnI, 0.1 to 10,000 pg/mL for BNP, and 1 to 10,000 ng/mL for Myo, and the LODs were well below acceptable limits for clinical application. However, a direct comparison with the conventional electro-chemiluminescence immunoassay has not yet been performed.

Pathogens are organisms (bacteria, viruses, fungi, parasites) that can cause diseases. They infect humans, plants, and animals through exposure to tainted food, contaminated water, and through the air. According to the World Health Organization (WHO), it is estimated that 10 million of people die every year due to bacterial drug resistance [40]. As of March 2022, severe acute respiratory syndrome coronavirus 2 (SARS-CoV-2), the causative agent of COVID-19, has accounted for more than 433 million infections and more than five million deaths worldwide [41]. The pandemic raised awareness of the importance of infection detection in order to prevent the spread of disease and to reduce disease risk. Therefore, the detection of pathogens is crucial for responding to specific problems, including outbreaks of new pathogens and the spread of antibiotic resistance.

Jia et al. developed a rapid detection method for two different strains of *Escherichia coli* by the assembly of enzyme-responsive chitosan hydrogel layers [42]. The fluorogenic substrate conjugated to the hydrogel reported the presence of enzymes β-galactosidase (β-Gal) and β-glucoronidase (β-Gus) for food-borne biosafety level 3 enterohemorrhagic *E. coli* and non-virulent *E. coli* K12, respectively, by a colour change at a single wavelength. The limits of detection of the highly stable sensing layers for an observation time of 60 min using a spectrophotometer correspond to the enzyme concentrations of β-Gus and β-Gal of ≤5 and ≤3 nM, respectively, and to ≤62 and ≤33 nM for bare eye detection in non-optimized sensor patches.

In a different approach, a similar *E. coli* detection strategy was developed [43]. Again, β-Gus was detected using a chitosan-based sensing hydrogel, this time coated on paper. The detailed analysis of the reaction kinetics was performed by a smartphone camera, which allows this sensor to be used as a POC test. In this chromogenic approach, a fluorophore was added by the well-established immobilization method using 1-ethyl-3-(3-dimethylaminopropyl) carbodiimide (EDS) and N-hydroxysuccinimide (NHS). In a two-step Michaelis–Menten reaction, a coloured response was afforded that could be translated into hue-saturation-value colour space and analyzed. The minimal time required for detection in this system is around 2 h at an enzyme concentration of 100 nM. The ratios of reaction rate and equilibrium constants of the enzyme substrate complex were about 0.3 and 0.9 pM/h for β-Gus in phosphate buffered saline and lysogeny broth, respectively.

**Figure 3:**
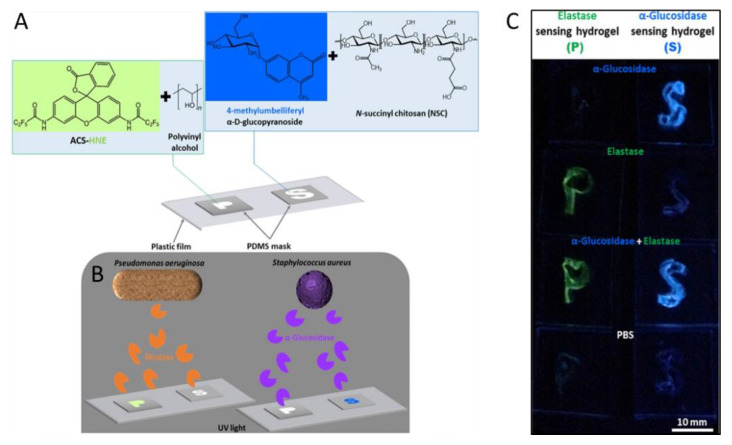
Schematics of the shape-encoded hydrogel sensors for detection and differentiation of *P. aeruginosa* and *S. aureus* (**A**,**B**). (**A**) Chemical structures of fluorogenic substrates and hydrogel matrices. (**B**) Fluorescence output in the form of the shape-encoded letters P (green) and S (blue) is defined by the corresponding target enzymes secreted from *P. aeruginosa* and *S. aureus.* (**C**) Photograph of the shape-encoded enzyme-sensing hydrogels after enzymatic reactions. The photo was taken after a reaction time of 60 min under UV illumination (hand-held lamp, λex = 365 nm) on a black background ([MUD] = 1 mM, [ACS-HNE] = 5 μM). Individual enzyme solutions, [α-glucosidase] = 1.0 μM and [elastase] = 10 μM, were added in the first and second row, respectively. A mixed enzyme solution ([α-glucosidase] = 2.0 μM, [elastase] = 20 μM with a volume ratio of 1:1) (Reprinted and adapted with permission from [45]. Copyright 2022 American Chemical Society).

Considerable research has investigated the detection of *Pseudomonas aeruginosa* (*P. aeruginosa*) and *Staphylococcus aureus* (*S. aureus*). These two microorganisms are considered clinically relevant multidrug-resistant pathogenic bacteria, that pose an important threat to public health due to the lack of reliable therapies [44]. Jia et al. developed two strategies to identify *P. aeruginosa* and *S. aureus* [45]. For *P. aeruginosa* a polyvinyl alcohol (PVA) hydrogel loaded with the fluorescent probe N,N′-(3-oxo-3H-spiro-[isobenzofuran-1,9′-xanthene]-3′,6′-diyl)bis(2,2,3,3,3-pentafluoropropanamide) (ACS-HNE) was designed that detects elastase, an enzyme encoded by the bacterium (Figure 3). The *S. aureus* detector used a chitosan-derived hydrogel loaded with the fluorescent probe 4-methylumbelliferyl-α-D-glucopyranoside (MUD). Again, an enzyme specific to *S. aureus* (α-glucosidase) posed as the analyte. The PVA hydrogel performed three orders of magnitude better than the related chitosan system. For an observation time of 60 min, LODs of ≤20 nM for elastase and ≤30 pM for α-glucosidase were obtained. Furthermore, both polymers designed were shaped in the form of the letters P and S for *P. aeruginosa* and *S. aureus*, respectively, to denote the two bacteria detected by the biosensors (Figure 3).

Wang et al. developed a hand-held quantitative assay for the detection of the main protease of SARS-Cov-2 in serum, using a self-made optical device [46]. For this, an optical cable linked to a laser pointer was ion-sprayed with gold and modified with a ruthenium catalyst containing a peptide probe for the noncovalent recognition of target proteins. The noncovalently bound protein was then photocatalytically cross-linked to form a gel, which can insulate the optical fiber leading to a positive read-out. A large dynamic detection range from 3.3 pM to 10 nM was observed, with an LOD of 1 pM.

### 2.2. Hormones and Metabolites

Hormones are small signaling molecules, which function to direct all aspects of development, function, stress response, and reproduction in living organisms [47]. Hormones also pose challenges as environmental contaminants as they interfere with ecosystems by altering the biochemistry of organisms [47]. There are many classes of hormones, including protein hormones, peptide hormones, and small molecule hormones. In humans and other animals, hormones are secreted by glands, where they enter the bloodstream and provoke regulative effects upon binding to specific receptors of the target tissue. Perturbations in the concentration of hormones in the body can lead to severe disease. Therefore, it is crucial for medical diagnostics and treatment to accurately determine the concentration of hormones present in body fluids [48,49]. However, conventional analytical methods such as chromatography are costly and time-consuming. Hormones are particularly challenging analytes to detect as they’re present in low concentrations in vivo (≤1 nM in blood) and often degrade rapidly outside of physiological conditions [47,48]. Given the challenges associated with detecting and quantifying hormones, hydrogels have emerged as a promising approach to detect hormones. Due to significantly enlarged surface areas, hydrogels possess high interaction capacities which enhance signal intensities [50]. There are efforts to further increase sensitivity and improve the suitability of hydrogel biosensors for the detection of hormones. Generally, optical transduction techniques including chemiluminescence (CL) and fluorescence are utilized for hydrogel hormone biosensors. Furthermore, an enhancement of signal intensities has often been achieved by modifying the hydrogel matrix with various nanoparticles that serve as fluorophores or exert catalytic effects.

Zhang et al. prepared a carboxylated p(NIPAM) hydrogel layer functioning as a waveguide for surface plasmons during the detection of the steroid hormone 17-β-Estradiol (E2) [51]. For this, bovine serum albumin (BSA) E2 conjugates were immobilized in a hydrogel layer. In a microfluidic approach, spiked test solutions were drawn over the sensor, which was able to detect free E2-antibodies by binding-induced refractive index changes. Compared to a regular surface plasmon resonance (SPR) biosensor, hydrogel optical waveguide spectroscopy (HOWS) achieved a seven-times higher detection limit (50 pg/L). However, the authors stated that an even higher sensitivity was limited by the slow diffusion of the large antibody through the hydrogel matrix. Addressing hydrogel properties such as pore size or polarity could improve the diffusion through the matrix.

E2 can also be detected by chemiluminescence sensors, as it has been shown by Fine et al. [52]. They enclosed a genetically modified yeast strain (*Saccharomyces cerevisiae*) into calcium alginate and PVA to produce luminescent signals induced by the binding of estradiol or corresponding endocrine disrupting chemicals (EDrCs). When treated with EDrCs, the cells express *luc* reporter genes that together with added D-luciferin result in a fluorescence signal. The authors highlighted the long-term stability of the yeast culture enabling repeated measurements without renewed cell cultivation.

A methodological concept for increasing the diffusivity of bulky hormones such as BNP was described by Mandon et al. They used a 3D-printing technique, called dynamic light processing (DLP), to arrange hydrogel-mediated peroxidase and Gox-based sensing layers in functional shapes [53]. The 3D-printing ink and PEG-700-DA derived structures were assessed with anti-BNP monoclonal antibodies and their treatment with the target molecule resulted in an intense specific signal. The use of a propeller-shaped hydrogel sensor with a rotation speed of 150 revolutions per minute (RPM) resulted in a five times higher chemiluminescent signal during BNP detection over no rotations.

Another biosensor employing chemiluminescence was prepared by Zhang et al. for the detection of human chorionic gonadotropin (hCG) in a sandwich immunoassay [54]. As a base layer, nanostructured poly(aniline) (PAni) hydrogel was provided with platinum nanoparticles, which foremost increased its conductivity and the antibody loading. Furthermore, Pt-NPs catalyzed the cleavage of H_2_O_2_ into reactive O_2_^•−^ and OH^•^ species, which promoted the oxidation of luminol and thus contributed to the increase of CL intensities.

Pourreza and Ghomi made use of metal-enhanced fluorescence (MEF) for the detection of insulin as such protein biomolecules possess intrinsic fluorophores [55]. For this purpose, they functionalized a PVA–Borax hydrogel using silver nanoparticles without the need for an additional reductant. MEF was achieved due to coupling interaction between fluorophore and plasmon resonance of Ag-NP if the distance between fluorophore and Ag-NP was less than 10 nm. Alongside signal amplification, the authors emphasized the biocompatibility of the hydrogel and the fact that the system did not require labels or reducing agents.

In contrast to this fluorescence turn-on mechanism, Bhuniya et al., exploited fluorescence quenching for the sensing of insulin [56]. Their simple synthesis method provided monosaccharide-based fluorescent gels and the gelators used showed strong gelation abilities even at low concentrations and self-assembling behaviour. One of the hydrogels based on a d-gluconolactone-pyrene derivative could detect insulin at concentrations as low as 5 μM. The monosaccharide–insulin interaction was not only detected by measuring the fluorescence quenching with fluorescence emission spectroscopy, but also by determining the layer thickness with scanning electron microscopy (SEM).

**Figure 4:**
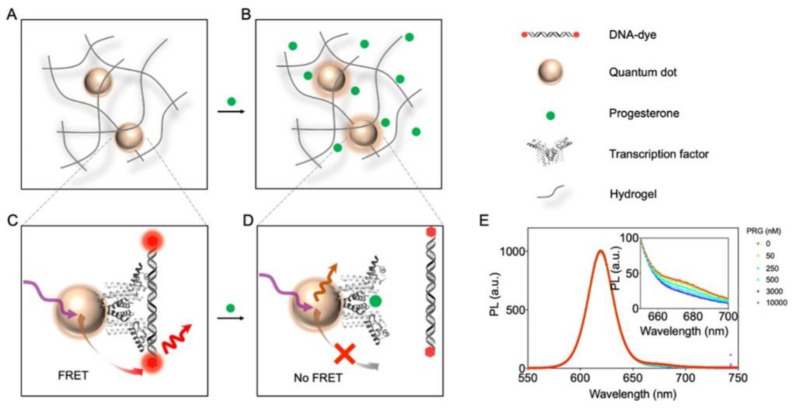
QD-based FRET sensor immobilized in the hydrogel. (**A**,**B**) Schematic illustration of progesterone diffusion into the hydrogel. (**C**,**D**) FRET-based sensor utilizing the TF–DNA binding mechanism. When progesterone (PRG) binds to the TF, the affinity between the TF and DNA reduces. The resulting dissociation of the TF–DNA complex produces a decreased FRET signal. (**E**) Representative spectra of sensor response to progesterone. A selection of the analyte concentrations is plotted for visual clarity. A zoomed-in figure is shown in the inset (Reprinted with permission from [57]. Copyright 2022 American Chemical Society).

Chen et al. also used fluorescence quenching sensors, but for sensing progesterone [57]. For the first time, a transcription factor was integrated as the bioreceptor into a PEG hydrogel that was attached to a quantum dot. Förster resonance energy transfer (FRET) between a fluorophore-labeled cognate DNA and the modified quantum dot (QD) was inhibited in presence of progesterone leading to a ratiometric attenuation of fluorescence intensity (Figure 4). With this study, the authors were able to demonstrate the potential of the novel genomics-to-sensor approach using allosteric transcription factors to overcome the problem of an insufficient number and integrability of adequate bio recognition elements. However, the relatively long dialysis time for progesterone removal was considered as a limiting factor, which needs to be improved upon in future work.

**Figure 5:**
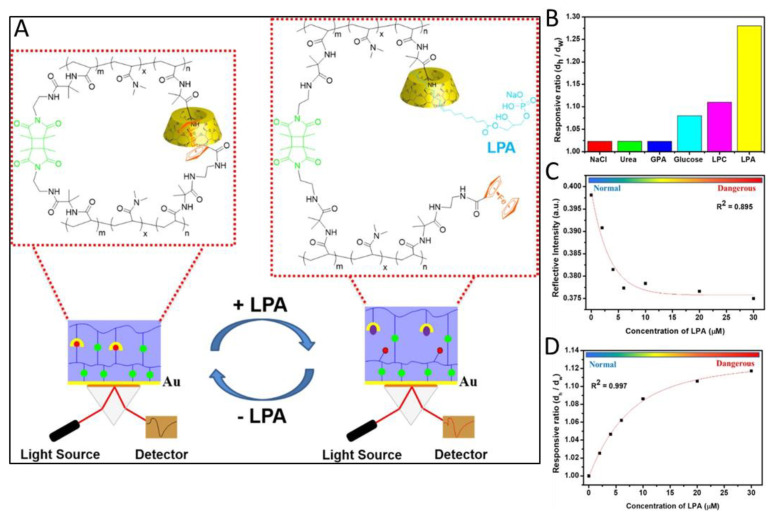
(**A**) Schematic illustration of the SPR-OWS sensor based on the dually crosslinked supramolecular hydrogel toward the specific detection of LPA in mimicked plasma conditions. (**B**) Responsive ratio (where d_h_ is the layer thickness of the hydrogel in diverse components and dw is the layer thickness of the hydrogel in water) of the DCSH in the presence of major blood plasma components. NaCl (100 mM), urea (5 mM), glucose (3 mM), GPA (10 μM), LPC (10 μM), and LPA (50 μM). (**C**) Plot of reflected intensity against the concentration of LPA in mimicked plasma from the measured SPR-OWS spectra at a certain angle of incidence of 57 and its nonlinear fitting. (**D**) Plot of the responsive ratio against the concentration of LPA from simulated parameters of SPR-OWS and its nonlinear fitting (Reprinted/adapted with permission from [59]. Copyright 2018 American Chemical Society).

Ovarian cancer shows one of the highest mortality rates compared to other types of cancer, since the symptoms related to it appear relatively late in its progression. One promising biomarker, lysophosphatidic acid (LPA), has been shown to be involved in the cancer initiation, growth, and metastasis of ovarian, prostate, and breast cancer cells [58]. One system capable of detecting LPA was presented by Li et al. [59]. The hydrogel system is based on a copolymer of dimethyl acrylamide, photocrosslinked via 3,4-dimethyl-maleimide. The dually crosslinked nature and cleavable crosslinks originate from host–guest complexes formed by β-cyclodextrine (β-CD) and ferrocene moieties within the polymer. Compared to other approaches in which the hydrogel only serves as a matrix or is formed by single crosslinks, which can cause irreversible degradation of the hydrogel after cleavage, the dually crosslinked hydrogel sensor presented in this system offers much higher mechanical stability. When exposed to LPA solutions, the competing complex formation of LPA with the β-CD led to swelling of the hydrogel through cleavage of the host–guest crosslinks (Figure 5). The β-CD-LPA complex showed 1:1 and 2:1 binding motifs in 1D nuclear magnetic resonance (NMR) and high resolution mass spectrometric (HRMS) measurements, with a complexation energy of −15.6 kcal/mol, determined by isothermal titration calorimetry (ITC). The evaluation of the test results was carried out by SPR measurements and showed great sensitivity across the concentration range of 1 µM to 50 µM, while the response was much more sensitive to LPA when compared to other targets such as glucose or lysophosphatidylcholine (LPC) which were also present in plasma samples (Figure 5).

Metabolites are particularly low molecular weight biomolecules (<1500 Da) that occur as intermediates or end products in cellular metabolism [60]. Metabolites can be of endogenic sources (e.g., cell processes) or of exogenic sources (e.g., diet and microbes) [61]. Hence, they assume important roles in energy conversion, signaling, and epigenetic regulation [61,62]. As metabolites are produced through physiological processes, they serve as biomarkers for a wide range of metabolic disorders. Common biomarker metabolites include ethanol, urea, lactate, glucose, and ammonia [16]. Changes in the concentration of these metabolites in bodily fluids can be in an indicator of serious diseases [63]. For example, high triglyceride levels are indicators for the increased risk of cardiovascular or coronary heart diseases [64], as high glucose levels are for diabetes mellitus and high bile acid levels are for liver or intestinal diseases [65]. Consequently, efficient health care, diagnostics, and treatment depends on the accurate determination of metabolite concentrations in the blood. Apart from elaborate chromatographic analysis, biosensors have been increasingly established in recent years owing to their user-friendliness and portability. Furthermore, current research has focused on the implementation of hydrogels in biosensors for metabolites because they can store larger amounts of target substances, thus increasing sensitivity. It is obvious that most of the biosensors exploit enzymes for recognition of the target substance since they exhibit high substrate specificity.

One outstanding example for an ammonia sensor is given by Liu et al. [66] who investigated the application of a hydrogel made from poly-L-glutamic acid (PLGA) and L-glutamic acid (LGA) to detect the presence of ammonia in exhaled breath [67]. They were interested in ammonia, as it has been identified as a reliable breath biomarker for kidney dysfunction. The ammonia reacts with the carboxylic groups in the hydrogel, forming ammonium ions and thereby changing its conductive properties. The change in conductivity is measured with electrodes and corresponds to the concentration in ammonia. Their results show a highly sensitive and selective system with the lowest detected concentration of 0.5 ppm ammonia. Unlike already existing detection systems, the presented hydrogel shows a much higher applicability when used under highly humid conditions, such as human breath samples.

Erfkamp et al. exploited the pH sensitive swelling of a poly(acrylic acid-co-dimethylaminoethyl methacrylate) hydrogel resulting from enzymatic hydrolysis of the analyte urea [68]. The enzyme was entrapped during the polymerization and showed full functionality in a simple urea conversion that was monitored with a pH probe. The swelling pressure related to the urea concentration was converted to a measurable voltage using a piezoresistive mechanism. The sensor possessed a broad linear range covering 0.001 to 20 mmol/L and allows ratiometric results. Furthermore, the authors emphasized the long-term stability of the sensor exceeding eight weeks without loss of enzyme performance, that can be increased by additional use of the standard EDC/NHS method. Lastly, the sensor showed exceptional specificity upon treatment with similar species (thiourea, *N*-methylurea, and *N,N,N’,N’*-tetramethylurea) as well as an in-line process capability.

Hasanah et al. developed a triglyceride biosensor using a colourimetric transduction method [64]. This was realized by equipping a pectin hydrogel with the enzyme lipase and a chromoionophore (ETH5 294). Enzymatic cleavage of the triglyceride induced protonation of the chromoionophore, which was concomitant with a change in pH and a change in colour from pink (deprotonated) to blue (protonated). The alteration of colour absorbance was then measured with a UV-spectrometer and translated to the triglyceride concentration. The authors highlighted the biocompatibility of the pectin hydrogel overcoming the issue of many synthetic materials in terms of deficient permeability and environmental harmfulness over the long term.

In contrast to chromatographic techniques, biosensors are mostly limited to the specific detection of one analyte. An effort towards the versatility of biosensors was provided by Li et al. as they designed a hydrogel-based amperometric sensing platform capable of determining not only triglycerides but also uric acid and cholesterol [63]. They modified a conductive PAni hydrogel with platinum nanoparticles and covalently attached the corresponding enzymes using the bifunctional compound glutaraldehyde. The platinum nanoparticles firstly served as catalytic centers for the oxidation of hydrogen peroxide, which was released through the enzymatic breakdown of the respective analyte. Secondly, the nanoparticles supported the transference of the generated electrons into a measurable current flow. This electrochemical approach enabled a remarkable response time of approximately three seconds, which surpasses that of earlier reported sensors employing different electrode materials such as single-walled carbon nanotubes, zinc oxide, or chitosan.

**Figure 6:**
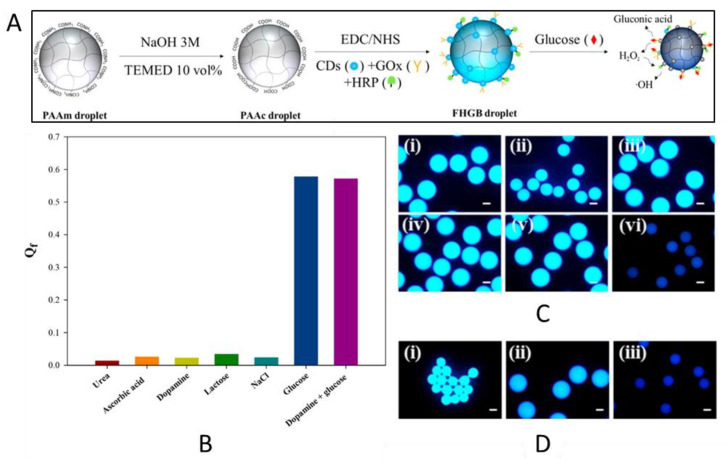
(**A**) Schematic of the preparation of the FHGB droplets. (**B**) Q_f_ of the hydrogel biosensor droplets (0.05 g) after (**i**) urea, (**ii**) ascorbic acid, (**iii**) dopamine, (**iv**) lactose, (**v**) NaCl, and (**vi**) glucose aqueous solutions (30 mM, 2 mL) are added to the cell, (**C**) fluorescence images of panel a, and (**D**) fluorescence images of the FHGB droplets in (**i**) the dried state, and (**ii**,**iii**) the swollen state in water, (**ii**) without, and (**iii**) with 30 mM glucose 4 weeks after drying; all scale bars in b and c are 200 μm; the PL spectra in panel a were obtained following 5 h of incubation (Reprinted and adapted with permission from [69]. Copyright 2018 American Chemical Society).

Another option for reducing the response time is to exploit microgels instead of more inert hydrogels. For example, Park and Park prepared hydrogel microdroplets exhibiting a dual-mode response in terms of fluorescence quenching and size reduction for the detection of glucose [69]. A cross-linked poly(acrylic acid) (PAAc) hydrogel was functionalized with carbon dots, Gox, and HRP from which microdroplets were obtained using glass capillaries with a coaxial flow-focusing geometry (Figure 6). Both fluorescence quenching and size reduction were initiated by the production of hydroxyl radicals and gluconic acid (pH reduction), respectively, as glucose underwent a reaction with glucose oxidase and horseradish peroxidase (Figure 6). According to the authors, this approach benefits from the usage of water-soluble and biocompatible carbon dots instead of other frequently employed quantum dots which are considered to be toxic, environmentally hazardous and more chemically unstable. In addition, due to the size of the microgel, the sensor is applicable to limited spaces where it delivers localized data rather than large-area averaged data.

Unlike the common exploitation of enzymatic bioreceptors, a sensor prepared by Deng et al. did not require a dedicated recognition element for the detection of bile acids namely cholic acid (CA) and deoxycholic acid (DCA) [65]. Their label-free approach made use of liquid crystal droplets which were distributed in chitosan hydrogel films. Their director configuration varied upon competitive adsorption of the probed facial amphiphilic analyte. The change in director configuration was transformed into concentrations using a polarization microscope. It was found that both the response time for DCA (30 s) was significantly lower than for CA (4 min) and the limit of detection for DCA (10 µM) was four-times lower than for CA (40 µM). The authors noted the suitability of the sensor for point-of-care diagnostics because of its high mobility, ease of use, and cost-effectiveness.

### 2.3. DNA Sensing

Deoxyribonucleic acid (DNA) detection has high importance in research due to its application in medical fields, where it is used for the diagnosis of gene mutations or diseases [70,71]. Biosensor-based DNA detection is achieved through measuring changes in electrochemical signal, calorimetric measurements, fluorescence, and pH changes [6,72,73,74]. Hydrogels are incredibly versatile for the detection of DNA as the gel is easily functionalizes with nucleic acids [75]. Compared to the two-dimensional hydrogel matrixes, the three-dimensional structure can capture more probes, thus improving sensitivity [76].

Kowalczyk et al. prepared an electrochemical DNA sensor by a dual-target hybridization assay on a poly(N-isopropylacrylamide-co-acrylic acid) gel [77]. For this, DNA marked with anthraquinone (AQ) and ferrocene (Fc) derivatives were immobilized by hybridization with hydrogel-bound single stranded DNA (ssDNA). This selective process allowed the detection of the targeted strands by electrochemical methods such as cyclic voltammetry (CV) and electrochemical quartz crystal microbalance (EQCM). Furthermore, laser ablation inductively coupled plasma mass spectrometry and optical measurements such as UV–Vis spectroscopy and circular dichroism were used to monitor the hybridization. The extensive investigation showed a nearly 100% hybridization of the target strands with the gel-bound ssDNA. The detector shows an exceptional LOD of 0.8 pM for DNA-1-AQ and 70 fM for DNA-2-Fc. It can easily be recovered by a cyclic potential, which makes it even more attractive for its use in immunoassays.

Label-free testing of DNA was made possible by Sun et al. through their impedimetric mitochondrial DNA detector [78]. A bionic hydrogel was created from fish sperm DNA and the semiconductor graphene oxide (GO) in a centrifuge tube, which allows higher control over the gelation than regular electrode coating. To finish the sensor, this gel was combined with poly(ethylenimine) (PEI) to increase the impedance signal. The sensor was able to distinguish between three sets of mutated complementary DNA containing either one, two, or three base-mismatches as well as non-complementary DNA. Samples from cancer patients were analyzed successfully using this sensor with a linear detection range from m 1.0 nM to 1 pM and an LOD of 0.569 pM, 0.525 pM and 0.552 pM for the three mutations, respectively.

Chen et al. developed three-dimensional nitrogen-doped graphene-Fe_3_O_4_ nanoparticles which formed a sensing film to detect DNA by electrochemical biosensing [79]. The hydrogel was constructed by reducing a mixture of GO and FeCl_3_·6H_2_O and deposited on top of a glassy carbon electrode. Immobilized probe ssDNA was used as the for the hybridization of the sample strands. Through measurements of the electron transfer resistance with EIS glassy carbon electrodes (GCE), GCE modified with a GO, 3D graphene hydrogel immobilized on GCE, and 3D graphene hydrogel-Fe_3_O_4_ on GCE were compared. This revealed that the 3D graphene hydrogel-Fe_3_O_4_ facilitates the electron transfer better than the other systems. In this study differential pulse voltammetry (DPV) was used to determine the hybridization with methylene blue as an electrochemical indicator. The sensor showed a high selectivity for complementary DNA in comparison to mismatched ssDNA and it was fully functional in complex biological fluids. The broad linear range from 0.01 pM to 1 µM and the low detection limit of 3.63 fM make this sensor an interesting candidate for clinical applications.

Other 3D hydrogels applied for DNA detection and quantification can be prepared from peptides. Peptide hydrogels have been widely used in biosensors due to their high stability, sensitivity, and easy preparation [80]. However, the main problem of the hydrogel substrate is the small pore size often on the nano-meter scale, slowing the diffusion of analytes [81]. Interestingly, the findings from Kivlehan et al., showed one possibility to solve the diffusion problem [82]. They developed a surface-attached PEGDA hydrogel for the detection of specific target DNA sequences such as the cystic fibrosis transmembrane conductance regulator (CFTR) gene. In their study, hydrogel structures were formed by a photopolymerization process, while the pore size is controlled by the PEGDA content in the prepolymer mixture. Amino-modified probe oligo sequences in the hydrogel were used for the hybridization of target strands. Epifluorescence microscopy was used to detect fluorescently-marked complementary strands after the hybridization. While the sensor was able to discriminate between wild-type and mutant allele sequences down to concentrations of about 1 nM, the test also shows elevated background fluorescence due to nonspecific binding. However, the short hybridization time of only 15 min makes this sensor a viable option for lab-on-chip or POC tests.

King and coworkers reported on, for the first time, fibre-bound DNA recognition motifs on a self-assembled 3D peptide hydrogel [83]. In their work, DNA aptamers that can hybridize a fluorescent molecular beacon sequence are conjugated to the self-assembling peptide. A fluorescence signal was produced upon the hybridization that showed an LOD of 22 pM, even under biologically relevant conditions. However, for future applications the molecular beacon has to be immobilized for a functional device.

Another study reported hydrogels formed by lithocholate (LC^−^) and metal ions that can serve as the precursors for producing a new network of CuS nanoparticles [84]. Specifically, the LC^−^/Cu^2+^ hydrogel showed a high fluorescence quenching ability in ssDNA detection. The precursor containing the facial amphiphile and Cu^2+^ was precipitated at room temperature with thioacetamide solution to form aggregated nanoparticles. Carboxyfluorescein-labeled ssDNA probes were detected by the efficient fluorescence quenching due to the CuS particles. The high interaction rate can be explained by the large surface area and porous network property of CuS nanoparticles. The linear range was 0 to 20 nmol/L with an LOD of 0.8 nmol/L, which is comparable to previously reported systems.

Mao et al., developed a method to visually detect and quantitate circulating tumor DNA (ctDNA), an important biomarker describing tumor dynamics [85]. The method relies on a ctDNA-triggered DNAzyme-functionalized hydrogel to generate signals. In detail, target ctDNA goes through rolling circle amplification and synthesizes a DNA hydrogel, which is large enough to be observed by the naked eye. Then the hydrogel catalyzes the colourless ABTS-H_2_O_2_ system to green through the DNAzyme activity. The quantitating of ctDNA could be achieved by detecting the colour change of the ABTS–H_2_O_2_ system. This work shows great sensitivity for ctDNA with a detection limit of 0.32  pM and has been successfully applied to the analysis of blood samples.

Zhao et al. developed a hydrogel suspension array consisting of quantum-dot-tagged DNA-responsive hydrogel photonic beads [72]. Hydrophilically-modified colloidal silica beads were used as a template for the copolymerization of *N,N’*-methylenebisacrylamide and 5′- and 3′-acryloyl-modified ssDNA. The CdSe QDs were added by EDC surface modification. Hydrogel shrinking can be observed when the target DNA hybridizes with the ssDNA in the hydrogel and the amount of DNA was estimated by the subsequent blue shift of the Bragg diffraction peak position. The hydrogel did not only have a large capacity of high-quality encoded microcarriers, but it also showed a high sensitivity with a detection limit of 1 nM, which is better than other expensive methods, such as SPR or QCM.

**Figure 7:**
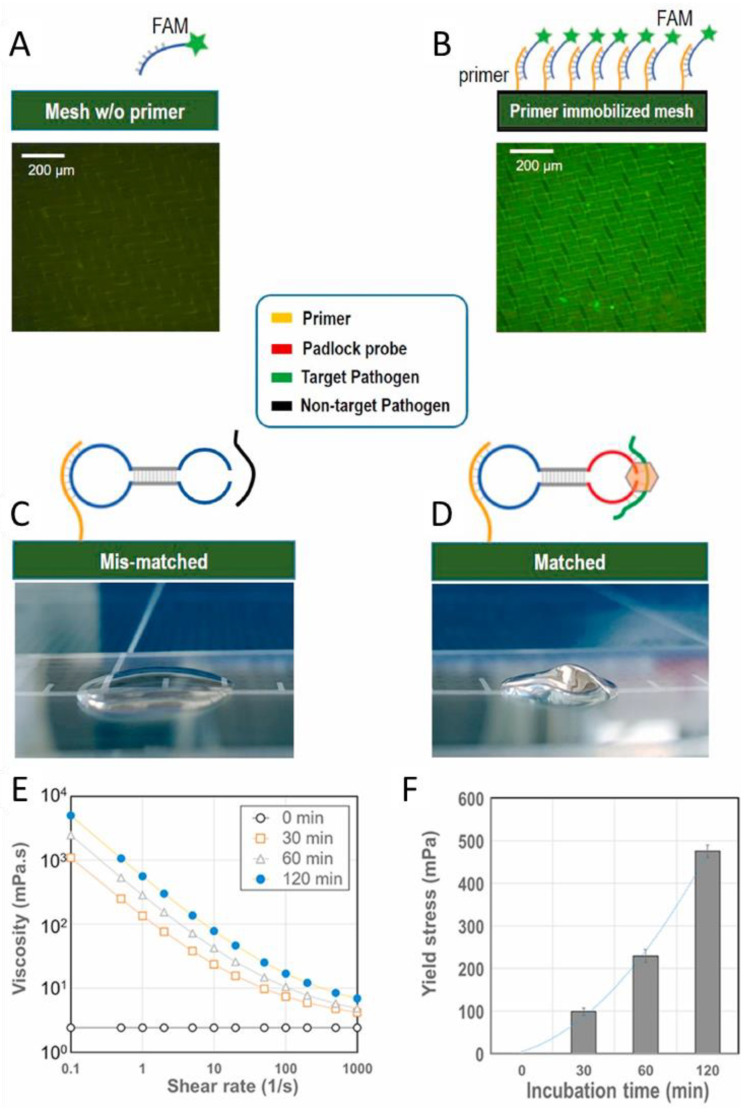
Validation of DNA gelation on a nylon mesh surface. (**A**) Schematic of the nylon mesh surface without primer immobilization and hybridization with the fluorescence probe FAM; a 200 μm fluorescence microscopic image is also shown (lower image). (**B**) Schematic of nylon mesh surface with primer immobilization and hybridization with the fluorescence probe FAM; a 200 μm fluorescence microscopic image is also shown (lower image). (**C**) Mobile phone-captured image of a mismatched experiment; a non-target pathogen (dengue pathogen) was used with the COVID-19 DNA template. (**D**) Mobile phone-captured image of a matched experiment; a target pathogen (COVID-19 pathogen) was used with the COVID-19 DNA template. (**E**) Increase in viscosity with incubation time (0, 30, 60, and 120 min) (n = 5), (**F**) Increase in yield shear stress with incubation time (0, 30, 60, and 120 min) (Reprinted from [87]).

Viruses can be detected by identifying their genetic material, as is performed with polymerase chain reaction (PCR) tests [86]. Kim et al. established a microfluidic point of care testing device for SARS-CoV-2 using isothermal amplification of nucleic acids on a nylon mesh [87]. In their methodology, ssDNA templates were self-assembled to form asymmetric dumbbell shapes that are immobilized by mesh-bound primers (Figure 7). Target ssDNA associated with the dumbbell and was ligated to form a closed-loop template. Rolling circle amplification was used to afford a DNA-based hydrogel. While a change in the hydrogel texture was visible, the detection was performed by the analysis of the fluid movement. The limit of detection reported as 0.7 aM after 15 min of incubation.

Wang and Li developed a quartz crystal microbalance (QCM) biosensor to identify an avian influenza virus (AIV), the highly pathogenic strain AIV-H5N1 [88]. An aptamer which displayed high affinity and specificity to AIV-H5N1 was used, and hybridization between the aptamer and ssDNA formed the cross-linker for the poly(acrylamide-*co*-aptamer) hydrogel. When no interaction between the biosensor and the virus occurred, the hydrogel remained shrunken, whereas when interaction occurred, an abrupt swelling of the hydrogel was observed. This was due to the reaction between the aptamer and AIV-H5N1 virus that caused the dissolution of the linkage between the aptamer and ssDNA. The QCM sensor detected this swelling mechanism by measuring the decrease in frequency. They showed a total detection time from sampling to detection of 30 min.

**Figure 8:**
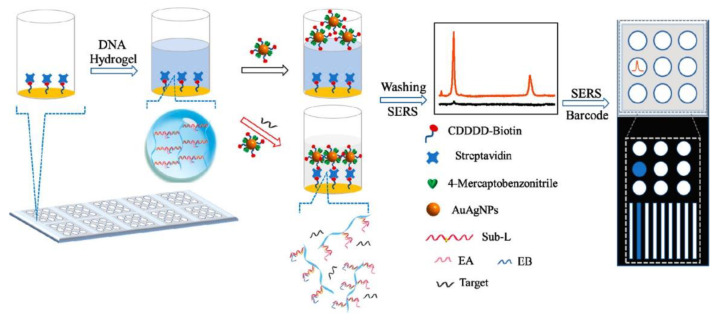
Schematic illustration of the preparation and application of the target miRNA-responsive DNA hydrogel-based SERS sensor array for measuring multiple miRNAs in one sample (Reprinted with permission from [91]. Copyright 2020 American Chemical Society).

One rather novel subgroup of biomarkers are micro RNAs (miRNAs), which are short noncoding RNA fragments of nucleotidic composition. Like proteins, these fragments play a vital role in the normal functioning of cells, especially as regulators in gene expression processes. Abnormal expressions of miRNAs are associated with the presence of a number of diseases including diabetes [89], various types of cancers, and certain neurological disorders, and they are therefore a measurable diagnostic indicator [90]. Since they were identified as reliable biomarkers, several hydrogel-based sensor detection methods have been established. While being reliable targets, the process of measuring miRNAs has proven to involve a manifold of laborious pre-detection steps and instrumentations [17]. The approach of versatile hydrogels again offers a novel and much easier measurement system. As an example, Wang et al. synthesized a hydrogel based on poly-acrylamide, containing two complementary DNA sequences serving as the stimuli responsive crosslinks between the different polymer strands. When exposed to samples containing the targeted complementary miRNA fragments (miR-21), the association to the DNA sequences leads to degradation of the crosslinks and therefore a visually observable cleavage of the gel. Remarkably the sensor showed an observable response for concentrations in the pico-molar range, while still showing the potential to be used as a point-of-care test [17]. A different DNA-based hydrogel approach was carried out by Si et al. [91]. Their system however is based on the coating of Au–Ag alloy nanoparticles for surface-enhanced Raman scattering (SERS). The hydrogel blocked streptavidin sensor units, which were liberated upon miRNA exposure. This allowed the nanoparticles to penetrate the hydrogel and to interact with the sensor surface to give a strong Raman signal (Figure 8). Sample preparation and data acquisition are more difficult compared to Wang et al. as their system allows for a much wider array of miRNA fragments to be detected but loses the POC test aspect in the process.

## 3. Drug Sensing

### 3.1. Sensing of Pharmaceutical Drugs

Rapid detection of pharmacological drugs has a number of critical applications, including measuring if dosages are correct, or detecting adulterants in common street drugs to prevent overdoses. Many drugs have a specific therapeutic window in which they are effective. Under-dosage could lead to negative consequences such as an insufficient supply of the drug, which in case of antibiotics could lead to resistances. In the case of over-dosage, poisoning symptoms or death could occur. In order to check the exact dosage, hydrogel sensors are an easy way to provide fast results without time-consuming preparation and evaluation. There are different types of sensors which can be used for the detection of drug molecules: non-imprinted recognitive hydrogels, molecularly imprinted hydrogels or biomolecule-containing hydrogels [92]. With surface modification or different detection method combinations, hydrogel biosensors can detect and quantify the active pharmacological ingredients fast, safely, sensitively, and selectively.

One type of hydrogel that has been particularly useful in sensing drugs is PhC hydrogels. PhC hydrogels integrate the swelling properties of hydrogels with the optical properties of PhC which allows for label-free detection of the analyte of interest [4]. Bile acids are used for the treatment of primary biliary cirrhosis and non-alcohol-induced steatohepatitis [93]. Wu et al. designed a self-reporting sensor based on molecularly imprinted photonic hydrogel (MIPH) for cholic acid. This method combines colloidal crystal template with molecular imprinting technique to prepare 3D-ordered porous hydrogels. For this, cholic acid was mixed with the methacrylic acid (MAA) and ethylene glycol dimethylacrylate (EGDMA) containing monomer solution on a silica crystal template. The mixture was polymerized between a photonic crystal template and a PMMA substrate to give a sandwich structure. After removal of the silica and cholic acid template, the sensor showed 3D-ordered interconnected macroporous arrays that improved the transport of analytes. Nanocavities provide high specificity owing to high substrate affinity, so the hydrogel sensor was able to discriminate between three bile acids. Detection was performed by observing the shift of the Bragg diffraction peak in relation to the analyte concentration. These sensors do not require any preparation of the analyte and the response time is as low as 60 s.

A similar approach was described by Meng et al. whereby they developed a convenient and effective method for the label-free and rapid detection of trace atropine in human urine [94]. The MAA–EGMA-based MIPH was designed to be water-compatible and showed a high sensitivity (as low as 1 pg/mL), fast response (less than 30 s), and specific detection of atropine in complex biological matrices. The detected concentration range of atropine is relatively wide, from 1 pg/mL to 1 mg/mL, contributing to the wide optical signal of colour change detected by the naked eye, which can be used for semi-quantitative analysis.

A highly specific and sensitive MIPH sensor was fabricated by Yuan et al. for the detection of 3-pyridinecarboxamide, also known as vitamin B3 [95]. The sensor provides a rapid and reversible visible response upon the detection of the target molecule. Through molecular imprinting, the hydrogel has very specific docking sites for 3-pyridinecarboxamide and subsequent swelling of the hydrogel results in a change of the diffraction peaks. The system can be adjusted by the size of the polystyrene spheres in the photonic crystal so that the change in the diffraction peaks is in the visible range. It is therefore possible to detect a colour change in the system with the naked eye without expensive equipment if the target molecule is present in the sample solution. Even with structurally very similar molecules, there are only slight changes in the diffraction peak that even at high concentrations do not lead to a drastic colour change of the system.

Because many sensors have limited surface immobilization capabilities, Li et al. proposed a dextran hydrogel-coated gold sensor chip for SPR analysis [96]. The gel was prepared by photo crosslinking dextran to a PEGylated gold surface to enhance the surface immobilization ability. The density of the dextran hydrogel was optimized, not only to immobilize proteins, but also to detect smaller molecules. The hydrogel’s protein immobilization capacity was ten times that of a bare gold surface and twenty times that of an 11-mercaptoundecanoic acid surface. However, first, the dextran sensor was modified by immobilizing the proteins hIgG, FKBP12, and streptavidin by the EDC/NHS method. Through this, the sensor was able to detect goat-anti-human IgG, the immunosuppressive drug FK506, and biotin in a microfluidic process. They observed an LOD of 0.5 nM and a high throughput detecting ability. Furthermore, they suggest that the dextran hydrogel-coated SPR technology can be combined with cutting-edge protein microarray technologies.

To effectively detect digoxin (DGX), a drug used against congestive heart failure, in human plasma samples, Shirani et al. have reported a simple and non-toxic aptamer sensor [97]. This is the first time that an aptamer-PVA-AuNP-fluoroprobe has been used for on-off-on fluorescence detection in a biosensor. Ultra-low levels of DGX are determined and time-consuming extraction and pre-treatment are not required in the actual sample analysis step. The AuNPs quenched the fluorescence of the PVA-hydrogel; however, it could be recovered by the addition of DGX by forming an aptamer/analyte conjugate. The analysis was performed with fluorescence spectroscopy. No significant interference from other prescription drugs or ions in plasma were observed. The sensor was also successfully applied in human plasma samples with minimal modification. The dynamic detection range of 0.01–1 mg/L met the requirements of the therapeutic dosage. Furthermore, in plasma a linear detection range of 10–1000 ng/L with an LOD of 3.1 ng/L were established.

Caffarel-Salvador et al. initially employed hydrogel-forming microneedle (MN) arrays for the minimally invasive extraction and measurement of pharmacological compounds and glucose from the skin in vitro and in vivo [98]. Compared with traditional blood sampling, MN can perform painless detection of drug molecules without irritating skin nerves or causing bleeding. The poly(methyl-vinylether-*co*-maleic anhydride) and PEG derived MNs were used to absorb the asthma drug theophylline, caffeine, and glucose upon skin injection. After removal, the gels were extracted, and the concentration was analyzed with HPLC. Several in vivo studies demonstrated that the MNs were highly biocompatible. These hydrogel MNs will facilitate therapeutic drug monitoring, replacing the current invasive blood sampling techniques.

Paracetamol, also known as acetaminophen or by its trade name Tylenol^®^, is one of the most commonly used non-opioid analgesics. Paracetamol overdoses are common and can cause liver damage and in severe cases result in death. Given this, it is important to develop methods for the fast and reliable detection and quantification of paracetamol in blood samples to determine if an overdose has occurred and gauge the severity. Different approaches for the detection of paracetamol using hydrogel sensors have been investigated. Bahram et al. utilized surface plasmon resonance of gold nanoparticles embedded in a hydrogel to detect acetaminophen, ascorbic acid, and folic acid [99]. When the sensor is exposed to these analytes, the hydrogel with the nanoparticles interacts with the substances, which enhances the surface plasmon resonance. The detection limit of the reported method is 1.8 µM.

Aside from using SPR for detection, electrochemical methods can also be used very reliably for the detection of acetaminophen. Herein, the systems are investigated using cyclic voltammetry or linear sweep voltammetry [100,101,102,103]. With an increasing concentration of the target molecules, a linearly increasing current could be observed in the cyclic voltammograms [103]. The detection limits of these electrochemically-used hydrogel systems were 1.8–3.0 nM. The articles published by Havens et al. and Kokulnathan et al. are particularly noteworthy in this regard. They fabricated hydrogels with embedded carbon nanotubes and graphene nanosheets, respectively [101,103]. The large surface area of these materials greatly improves the detection of the target molecules.

Carboxylates are widely used in many industries, but their use as nonsteroidal anti-inflammatory drugs (NSAIDs) brings a significant environmental burden. Using principal component analysis and linear discriminant analysis, in 2013, Liu et al. proposed a supramolecular sensor array made up of octamethylcalix [4] pyrrole sensors-doped polyurethane hydrogel films that can detect carboxylate pharmaceuticals [104]. It has a highly accurate identification of 14 carboxylates in water and complex matrices as human urine. The multiwell array can distinguish six typical non-steroidal anti-inflammatory drugs (diclofenac, flurbiprofen, ibuprofen, salicylic acid, ketoprofen, and naproxen) from 0.5 to 100 ppm. For the commonly used NSAIDs, an LOD of 0.1 ppm was found. Fluorescence spectroscopy was used to detect anion-induced intramolecular partial charge transfer signals.

Another naproxen detector was developed by Laishram and Maitra [105]. They created a simple method for the detection of not only naproxen, but also propranolol, the first beta-blocker, by using Tb^3+^ luminescence in a Tb^3+^ cholate hydrogel. The gel medium removed the water molecules bound to Tb^3+^, which quenched the luminescence but also made the system rigid to minimize the non-radiative decay effect. Naproxen and propranolol could sensitize Tb^3+^ by ligating it and therefore lead to the fluorescence detection. The detection limit is lower than the ppb level, which represents the fact that this approach can be achieved with commercial tablets and serum samples without any pre-treatment. This technique can also be used to distinguish similar drug pairs such as propranolol/atenolol and naproxen/ibuprofen.

**Figure 9:**
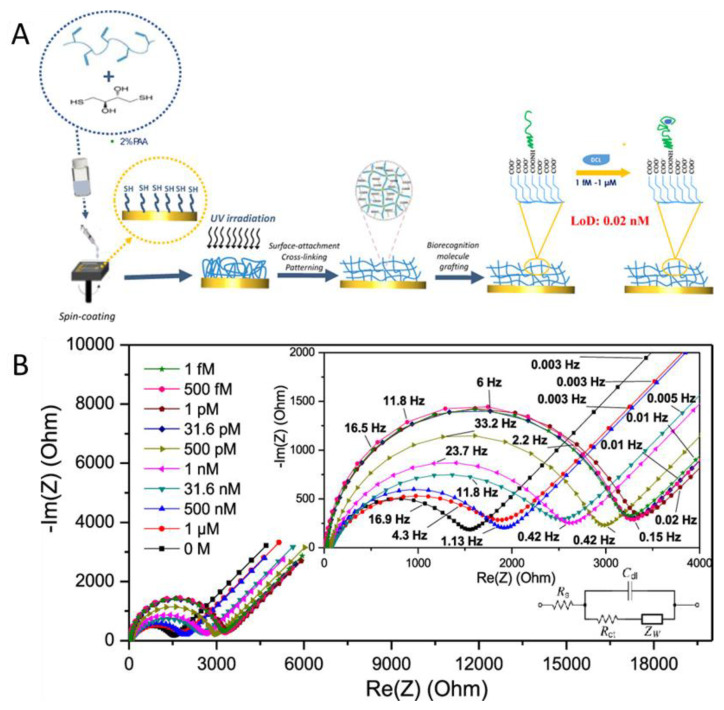
(**A**) Schematic representation of hydrogel preparation and sensing mechanism of DC. (**B**) Nyquist plot obtained in 0.1 M PBS containing 5 mM K_4_[Fe(CN)_6_] and K_3_[Fe(CN)_6_] after treatment of the aptasensor with different concentrations of DCL in the frequency range of 100 kHz to 1 mHz (Reprinted with permission from [106]. Copyright 2020 American Chemical Society).

The release of drugs into the environment and especially water is a major problem as even low concentrations of these pharmacological substances can have a huge impact on organisms. The easy and reliable detection and removal of these substances is an important task in wastewater treatment. One drug that is in focus in this regard is the NSAID diclofenac. One of the issues is the pharmaceutical drug diclofenac, known under the trade name Voltaren^®^, used to inhibit inflammatory diseases [106]. Diclofenac contamination in water has an impact on fish health [107]. Kassahun et al. focus on the development of a hydrogel aptasensor for the molecular detection of diclofenac using EIS [106]. The aptasensor consists of a gold electrode onto which a polyacrylic acid hydrogel layer is attached by means of thiol modification of the gold surface and grafting (Figure 9). The aptamers are subsequently covalently grafted to the hydrogel and are the recognition unit of the system for the detection of the diclofenac molecule. Their goal was to have a less expensive and more specific sensor than other reported electrochemical methods. Kassahun et al. obtained a layer thickness up to 57 nm in dry conditions. In water, the hydrogel had swollen to 1.8 times the size of the dry hydrogel. Nyquist plots obtained from the measurements are evaluated to confirm the presence of diclofenac in liquid samples (Figure 9). For the electrode in the diclofenac-free solution, the charge-transfer resistance (R_ct_) has a high value. This is due to the negatively charged aptamers on the electrode surface which inhibit electron transport. Upon the contact of the electrode surface with the diclofenac molecules, the negative charge density is reduced and R_ct_ decreases. The electrochemical signal is directly interpreted as a concentration. A linear dependence of R_ct_ on the concentration was found for the system in the range of 30 pM to 1 μM; the calculated detection limit of the electrode is 0.02 nM. The sensors provide a reliable reproducibility with a standard deviation of 4.8% over five tested aptasensors. The use of aptamers in combination with hydrogels for a sensing system is a simple way to enable the detection of specific molecules. This presented system allows simple and fast detection of drug residues, and for the evaluation of the results, no expensive or complex pieces of equipment are needed. An immobilization matrix for nanoscale measurements of diclofenac in freshwater environments could be efficiently provided for the first time.

The use of reduced GO-based hydrogels for the detection and adsorption of naproxen, ibuprofen, and diclofenac is a promising method explored by Umbreen et al. [108]. The interaction of the hydrogel with the target molecules depended mainly on electron donor–acceptor interactions, π–π interactions, and hydrogen bond interactions. In this context, the researchers investigated the influence of the pH on the system. They stated that the optimal pH range for the adsorption of the investigated drug molecules is between pH 2–3.5; a higher pH would result in an electrostatic repulsion between the analytes and the hydrogel surface. The porous structure of the hydrogels promotes the fast adsorption kinetics; an equilibrium is established within 60 min. The removal efficiency ranged from 65% for naproxen to over 80% for ibuprofen and diclofenac, which are promising results for the future use in water purification. Since environmental samples contain many different salts, the influence of NaCl content in the samples was investigated. Small amounts of NaCl, up to 15% for naproxen, enhanced the removal efficiency. The researchers hypothesized that this enhancement in efficiency is due to an increase in osmotic pressure, causing higher adsorption. NaCl concentrations higher than 15% led to a decrease in removal efficiency, which the researchers contributed to the ions blocking the binding sites of the hydrogel.

As already covered in the previous section, sensor hydrogels can be used to release embedded molecules after the recognition of a release-triggering drug molecule. The opposite mechanism can also be applied. It is possible to produce hydrogels, which, upon sensing a specific drug molecule, can adsorb it onto their structure and can therefore be used to remove them from the environment. This approach was used in two articles by Feng et al. and Godiya et al. for the development of hydrogels which can remove diclofenac from water [109,110]. For both investigated systems, the recognition of the target drug molecule is based on three interactions. Electrostatic forces between the negatively charged diclofenac molecule and the positively charged amine groups of the hydrogel. Hydrogen bonding and π-π stacking further enhance the adsorption. Feng et al. used a chitosan–genipin hydrogel system with embedded carbon dots to enhance the adsorption percentage to almost 100% for a 0.01 mg/mL diclofenac solution after 5 h [109]. Godiya et al., prepared an egg albumin–polyethyleneimine hydrogel which under optimum conditions shows an adsorption capacity of up to 232.5 mg/g [110]. Both systems are interesting candidates for use in wastewater treatment as they are entirely biobased materials, easy to fabricate, and do not need additional expensive equipment. The hydrogel prepared by Godiya et al. also showed a limited recyclability over about 4 cycles which is another advantage of this system [110].

Sensing for antibiotics is important in the quality control of agriculture and food and ultimately our health. Consuming too many antibiotics can have toxic effects on the body and presents a risk of developing drug resistance. Using hydrogels to sense antibiotics is attractive because they can be made to be biocompatible and responsive to a variety of stimuli [111]. This allows them to be used not only in the body, but also in environments such as in our drinking water [112] and in our food [113].

**Figure 10:**
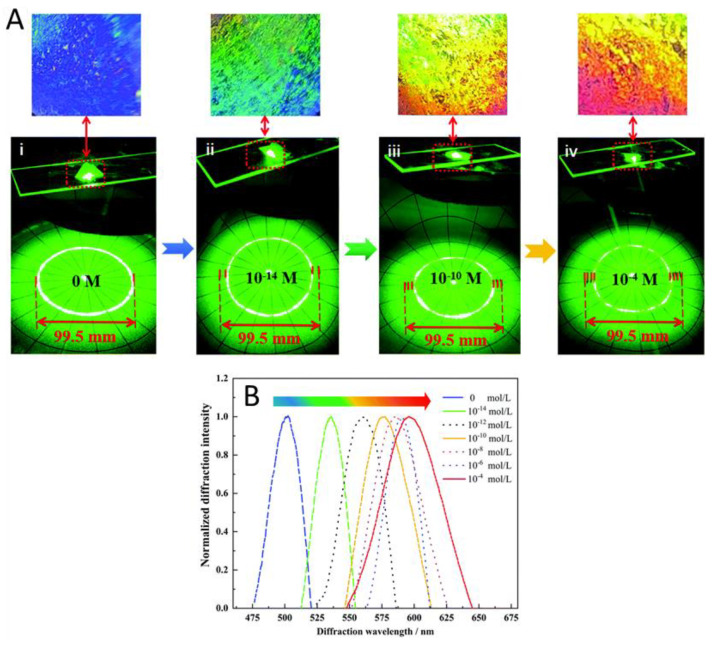
(**A**) Changes in the Debye ring diameter and colour of the SMZ–MIPCH sensor at different SMZ concentrations. (**B**) Dependence of the normalized diffraction spectra of SMZ–MIPCH on the SMZ concentration. (The measurement angle between the probe and the normal range to MIPCH is 21°) (Reprinted with permission from [114]. Copyright 2021 Royal Chemical Society).

A 2D molecularly imprinted photonic crystal hydrogels (MIPCH) sensor was made by Cao et al. in order to sense sulfamethoxazole (SMZ) [114]. Their sensor was relatively similar to the previous example except their binding pocket was made specifically for SMZ. They showed that they were able to obtain an increase in wavelength at SMZ concentrations as low as 0.01 pM, with a complete red shift being obtained at 0.1 mM SMZ (Figure 10). They also showed that the 2D MIPH had high selectivity for SMZ and not its analogues which is made possible by the SMZ-specific binding pocket. Finally, they tested their sensor in both pure water and simulated water samples for the detection of SMZ. They were able to obtain an LOD of 0.1 fM in both sample conditions, showing that 2D MIPH sensors can be made to be highly sensitive and selective.

Two-dimensional PhCs can also be integrated with ternary complexes to improve the swelling of the hydrogel. Zhang et al. added tryptophan (Trp) and Zn^2+^ to a PhC hydrogel to allow for an enhanced red shift because of an additional increase in osmotic pressure [115]. The indole group on Trp is left outside of the polymer chain to bind to Zn^2+^ which creates a localized positive charge. This increases the osmotic pressure within the hydrogel matrix which in turn increases swelling and the diffraction wavelength. When ciprofloxacin complexes with Trp-Zn^2+^, there is a further shift in osmotic pressure which causes even more swelling and an increase in the diffraction wavelength. They were able to obtain a LOD of 0.05 nM. Comparing their results to the other articles, the use of ternary complexes does not seem to increase sensitivity to a large degree. When they applied their method to a ciprofloxacin-spiked eye drop sample, they obtained a linear range from 0.1 nM to 0.1 mM and successfully quantified the spiked ciprofloxacin in the sample

Another example of a 2D MIPCH by Wang et al. was used to detect oxytetracycline (OTC) in milk samples [116]. They embedded a 2D PhC array into a poly(acrylamide-*co*-acrylic acid) (PAM-AA) hydrogel film and eluted an OTC template onto the hydrogel to create OTC-specific binding pockets. When the OTC binds, this causes an increase in osmotic pressure, which causes the hydrogel to swell and increases the particle spacing of the PhC. Increased particle spacing can be related to a decrease in the Debye diffraction ring diameter, a relation that is unique to 2D PhCs. The increase in particle spacing increases the wavelength. The hydrogel was able to selectively bind to OTC with high specificity compared to other tetracycline (TC) analogues due to the unique functional group positioning of the OTC which would perfectly fit the binding pockets. They also tested their sensor in a milk sample that had been spiked with 60 μM of OTC. It was able to detect OTC in the milk with a similar particle spacing and colour shift as an aqueous sample with 60 μM OTC. The LOD in water was 5.4 mM while in milk it was 9.6 mM, which is not as low as other methods.

Aside from PhC hydrogels, there are numerous other ways to detect antibiotics in the environment, such as fluorescence. GO hydrogels are a good platform for creating antibiotic sensors. Tan et al. prepared GO hydrogels that utilized adenosine and fluorescein amidite (FAM)-labelled OTC aptamer probes as cross-linking agents between the GO sheets because of strong π–π interactions [117]. The π–π interactions between the aromatic groups of the GO and the nucleotides also allow GO to be an excellent quencher for the fluorescence of the aptamer; in the presence of the antibiotic target, the aptamers would preferentially bind to the antibiotic resulting in weakened π–π interactions [118]. This would cause the target–aptamer complex to be released from the hydrogel which would increase the distance between the aptamer and GO, resulting in a drop in FRET and fluorescence is emitted. Using pure water samples spiked with OTC, the researchers found that a GO hydrogel with 10 μM of OTC aptamer probes gave the best linear range of approximately 25–1000 μg/L and a limit of quantitation (LOQ) of 25 μg/L while also having an appropriate amount of mechanical strength. They were also able to obtain good recovery in OTC spiked in real water samples. They tested their method using a sulfadimethoxine (SDM) aptamer and they were successful in detecting SDM in pure water and in river water samples.

On other hand, the quenching of fluorescence can also sense antibiotics. Chitosan hydrogels are biocompatible and act as a structural platform for functional polymers because of electrostatic interactions and hydrogen bonding [119]. Malik et al. synthesized poly[5,5′-(((2-phenyl-9H-fluorene-9,9-diyl)bis(hexane-6,1-diyl))bis(oxy))diiso-phthalate] sodium (PFPT), a conjugated polyelectrolyte, and functionalized chitosan hydrogels with PFPT in order to sense tetracycline by fluorescent quenching and remove them by adsorption [120]. When the negative charges of PFPT interacted with the positive charges of chitosan, they formed a fluorescent film. When tetracycline is present, it will interact with the PFPT-chitosan film particularly between the −OH, −CONH_2_, −N(CH_3_)_2_ groups of tetracycline and the carboxylate groups of the PFPT. This allows electron transfer to occur and thus the fluorescence is quenched and the emission is blocked. They found that the LOD of the PFPT towards tetracycline was approximately 14.35 nM (6.80 ppb). Using a PFPT-chitosan film with 15% PFPT showed the greatest change in fluorescence as tetracycline was introduced and it was able to bind to 3.12 mg/g of tetracycline compared to 0.68 mg/g using a blank film.

Due to the low cost and high prescription rate, the output of tetracycline to the environment is leading to problems. For the determination of TC, Ehtesabi et al. provided an alginate hydrogel doped with carbon dots (CDs) [121]. The CDs act as an emitter of fluorescent light while the hydrogel is a solid immobilization matrix for the organic molecules. TC acted as a complexation substrate for the CDs and this led to a reduction of the emitted fluorescent light. They established a linear range from 1 µM to 20 µM with an LOD of 2 µM. While the adsorption of TC was a rapid process, the specific mechanism for adsorption has not been elucidated. One more example for the detection of TC was given by Luo et al. with a chitosan-based hydrogel containing CDs [122]. It achieved a LOD of 0.12 µg/L with a linear range of 0.2–1 µg/L. The hydrogel even had the highest possible TC-loading from other references with 541.3 mg/g.

There are also sensors that can detect antibiotics, which in turn triggers the release of other compounds. Ehrbar et al. developed a hydrogel that releases embedded biopharmaceuticals after being exposed to novobiocin [123]. The fabricated hydrogel consists of three components: a polyacrylamide backbone attached with bacterial gyrase subunit B (GyrB) and coumermycin. Coumermycin can form bonds with the two GyrB units which causes the gelation of the hydrogel. In this study, the vascular endothelial growth factor (VEGF) VEGF_121_ is embedded in the hydrogel. Upon exposure to the antibiotic novobiocin, the hydrogel dissolves and releases the embedded VEGF_121_. When novobiocin replaces coumermycin, it can only bond to one GyrB unit, which weakens the hydrogel network, causes it to dissolve, and allows the controlled release of the embedded substance. The dependence of the VEGF_121_ release on the novobiocin concentration was investigated. At concentrations of 1.00 mM, a rapid release of VEGF_121_ occurred and a plateau was reached within 10 h. At 0.25 mM, it took more than 30 h to release the same amount of VEGF_121_. This confirms the concentration-dependent release of the embedded substance and makes this system an interesting approach for controlled drug delivery using drug-sensing hydrogels. Overall, the development of antibiotic sensing using hydrogels is vast, with hydrogel biosensors demonstrating high sensitivity and selectivity for detecting antibiotics of interest.

### 3.2. Sensing of Illicit Drugs

**Figure 11:**
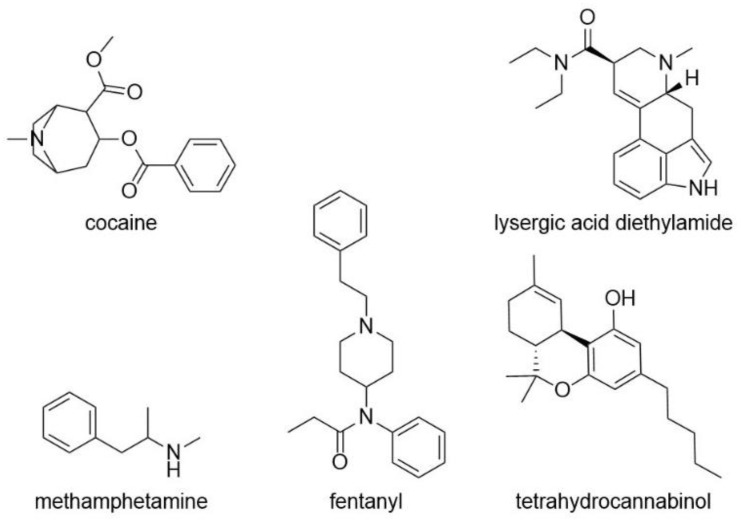
Common drugs structures that are discussed regarding hydrogels for sensing in this section.

The use of illegal drugs has been increasing steadily [124,125], particularly in the last two years of the COVID-19 pandemic [126,127]. Drug overdose is a serious issue that can lead to permanent health damage and in the most extreme cases death [128]. Here, we investigate hydrogel-based biosensors to detect some commonly used illicit drugs, including cocaine, fentanyl, and lysergic acid diethylamide (LSD), as well as anabolic steroids (Figure 11). Other compounds, such as tetrahydrocannabinol (THC), or ethanol are legal in certain jurisdictions [129,130,131]. All these compounds can be detected and quantified using hydrogel-containing biosensors.

The abuse of doping substances such as anabolic steroids and human growth hormones (hGH) is very common in sports. These doping substances increase sport performance but are also dangerous for human health and usually directly violate the sports’ rules [132,133,134]. To detect these kinds of drugs, urine or blood can be the used as a sample. One possibility to determine hGH is by detecting biomarkers in urine. The disadvantage can be that the biomarkers are present in low concentration and can additionally degrade quickly. To overcome these problems, NIPAM–AA hydrogel nanoparticles functionalized with Cibacron Blue F3G-A (CB) have been applied [134]. CB is a monochlorotriazinyl functionalized biomimetic dye and contains an anthraquinone chromophore. In several studies, CB was able to bind proteins and be used for affinity chromatography [135]. In this system, it is used as a ligand for both recombinant and natural hGH. The urine sample is mixed with the nanoparticles and they capture hGH. Afterwards, the sample is centrifuged and eluted in a standard clinical Immulite 1000 assay [134].

**Figure 12:**
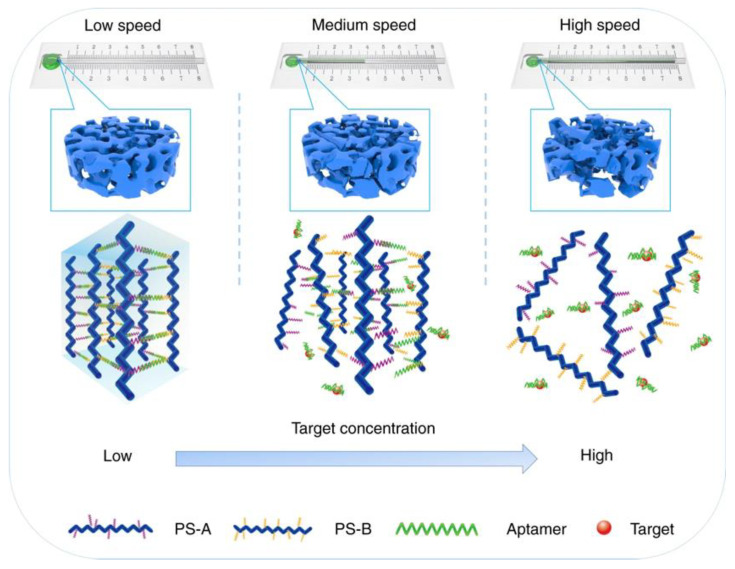
The working principle of the CSDR-Sensor. The addition of target will result in the degradation of hydrogel to a certain degree and then change the permeability of gel in the capillary tube and regulate the capillary action via membrane permeability (Reprinted from [137]).

According to Werner and Hatton, cocaine can also be used as an enhancement drug in sports and it was estimated to have 20 million users worldwide in 2019 [133,136]. Li et al. introduced an innovative method that utilizes a thermal reversible principle of DNA hydrogel for cocaine sensing in their capillary self-driven regulator sensor (CSDR-sensor) [137]. This sensor facilitates a linear relationship between the permeability of the capillary, thus the time the analyte is flowing through the probe, and the concentration of an analyte. An aptamer-crosslinked hydrogel based on acrylic-DMT phosphoramidite and two types of cocaine DNA strands was used to fabricate the sensor (Figure 12). The group reports an LOQ as low as 1.17 nM in a urine sample. Due to the low amounts (0.01 μL) of hydrogel needed for this test device, it is worthwhile for POC tests.

A different principle of detecting cocaine with the use of an aptamer cross-linked hydrogel structure was introduced earlier by Zhu et al. [138]. They utilized a cocaine aptamer developed by Liu et al. [139] and constructed a polyacrylamide hydrogel crosslinked using two DNA strands and a linker-aptamer strand that is complementary to the two strands. This gel structure held amylase enzymes, which were added to the mixture prior to cross-linking. When cocaine is introduced to the prepared gel, it competed to bind the linker-aptamer and this resulted in the gel dissolving. This step caused the release of the amylase previously entrapped in the structure. The enzyme reacted with present amylose and I_2_ changing the colour of the solution. The group reported to be able to visually detect less than 20 ng of cocaine [138].

Fentanyl is one of the most important opioids used for pain-management [140]. The abuse of this medication has increased by over threefold since 1990 and has led to an epidemic in the US [141]. Barfidokht et al. created an electrochemical glove-based sensor, which can rapidly detect fentanyl on-site [142]. This technology enables screening of fentanyl in the presence of a mixture of cutting agents. The glove-based sensor features a 3 mm hydrogel layer made from multiwall carbon nanotubes and an ionic liquid. After drop-coating flexible carbon electrodes with the mixture and placing them on a glove, fentanyl could be detected using the thumb (sampling) and index (sensing) fingers. A useful dynamic range of 10 to 100 μM was established, and the gel showed a high stability over two weeks under refrigeration. However, the testing system is for one-time use only, as the glove is contaminated afterwards.

There is a newer version of fentanyl, its analog butyryl-fentanyl. The two compounds are almost identical except for a small difference in the length of the carbon chain moiety. Liu et al. developed a fibre-optic sensor, which can measure the major metabolite of butyryl-fentanyl, carboxyl-fentanyl, in blood samples [143]. This sensor also has a potential to detect fentanyl due to the same binding sites to carboxyl-fentanyl. The group designed a long-period fibre grating (LPG) sensor array, which operates at phase matching conditions. One LPG array is coated with nano-sized molecularly imprinted polymers (nanoMIPs), which were synthesized via a solid-phase approach. Carboxyl-fentany was coupled to glass beads by the EDC/NHS method and a complex monomer mixture containing, among others, MAA, 2-hydroxyethyl methacrylate (HEMA), and styrene was polymerized on the solid support. In general, an LPG array is divided in two parts: the detection part, where the LPG array is functionalized with nanoMIPs, and the reference part. Here, the temperature will be detected since LPG sensors show a strong dependence on temperature variations. The detection of carboxyl-fentanyl ranged between 50–1000 ng/mL. The sensor was successfully tested in human serum samples without the interference of other small molecules such as glucose, BSA, or incidental drugs such as cocaine or morphine.

A well-known potent psychotropic drug is lysergic acid diethylamide (LSD). LSD can be detected in samples of urine or blood only for a few days (30–120 h) after it is introduced into the system [144]. An alternative to those two detection methods can be a hair sample, since the period over which LSD can be detected in hair is longer than in blood and urine. Chapuis-Hugon et al. synthesized a molecularly imprinted polymer (MIP) for the selective extraction of LSD. The group established a selective procedure while studying the interactions of LSD and MIP. However, for a low concentration of LSD, it is essential to obtain a qualitative assurance. For instance, Chapuis-Hugon et al. detected 0.1 ng/mg of LSD in hair with 82% extraction recovery and in urine at only 0.5 ng/mL with 83% extraction recovery.

Methamphetamine, also known as crystal meth, is a powerful psychostimulant and a derivative of amphetamine, a common attention-deficit/hyperactivity disorder (ADHD) medication. It is highly addictive, with 33 million estimated users worldwide [145]. Several sensors were developed as alternatives to the standard instrumental laboratory techniques for methamphetamine [146,147,148].

One example of a hydrogel being used was presented by Souza et al. [149]. Their agarose gel contained AgNPs to determine the concentration of aqueous methamphetamine solutions via surface-enhanced Raman scattering (SERS). The aggregated NPs form hot spots where the electromagnetic field is particularly intense, which allows even single-molecule SERS. The adsorption of the analyte induces a red shift in the spectrum, allowing detection down to a concentration of 0.1 µmol/L. Furthermore, they investigated the possibility of detecting methamphetamine transferred to a gel film by a finger. For this, a dilute methamphetamine-doped finger was pressed to the detector surface. Using this method, 190 µg of the drug could be detected, with a long-term sample stability.

Tetrahydrocannabinol (THC) is the main psychoactive component of *Cannabis sativa* L. [150]. *C. sativa* is cultivated mainly for its THC-rich flowers, which are used in recreational cannabis use. An electrochemical THC-sensor was introduced by Zhang et al. [151]. They used an MIP composed of cross-linked MAA-EGDMA monomers with THC as a template. The prepolymer solution containing these monomers was polymerized in carbon beads or carbon nano tubes and the THC molecules were removed by methanol, which created a polymer that can bind THC with high specificity. To determine the THC concentration ΔI was measured with differential pulse voltammetry. The authors reported LODs for the beads and the nano tubes of 0.32 ± 0.02 ng/mL and 0.18 ± 0.02 ng/mL, respectively. Like the methamphetamine sensor, the THC sensor showed remarkable stability.

**Figure 13:**
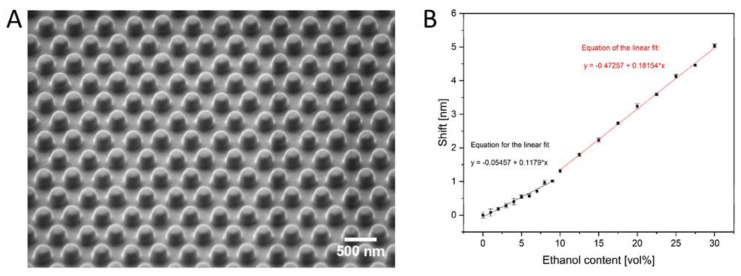
(**A**) Scanning electron microscope image (SEM) of the nanostructured gold sensor substrate. (**B**) Shift of the resonance wavelength with increasing ethanol content due to the deswelling of the PAM hydrogel showing the linear behaviour of the refractive index change (Reprinted from [152]).

Ethanol, if consumed frequently in high quantities, is harmful to human health. In addition, chronic consumption of alcohol can lead to addiction [131]. Kroh et al. presented an in-line monitoring of ethanol concentration during a brewing process that utilizes a hydrogel-based plasmonic sensor substrate, based on polyacrylamide and bisacrylamide [152]. The swelling and shrinking of the hydrogel upon ethanol treatment can be transduced by LSPR as it has been described before. Moreover, the authors reported the measurement range from 0 to 30 vol% ethanol with a response time of 5.2 min (Figure 13). Another benefit was the long-term sensor lifetime and the signal stability of ten days that corresponds with a regular brewing duration.

Ethanol vapor can be sensed with stimuli-responsive hydrogels, which can be employed as breathalyzers. Here, a chemical compound of the hydrogel network can swell significantly when the sensor is exposed to ethanol vapors. The concentration of ethanol can also be determined by an optical characterization of hydrogel-infiltrated photonic crystals [153,154]. For this, Dispenza et al. developed a HEMA–AAc copolymer that was crosslinked using (PEG_200_-DMA) [153]. HEMA was used due to its favorable Flory–Huggins mixing parameter with ethanol. Polystyrene colloidal crystals were incorporated, so that a swelling would result in Bragg diffraction peak shifts in the periodically ordered hydrogel film, as has been described before. Remarkably, the hydrogel inverse opals showed a reversible, visible colour change from iridescent green to red. On the downside, the dwelling time (220–240 min) of the ethanol vapor on the hydrogel is too long to be of practical use as a breathalyzer.

## 4. Hydrogel-Based Sensors for the Detection of Environmental Pollutants

Environmental sensors are developed and used for the measurement, monitoring, recording, and assessment of environmental parameters such as temperature, humidity, gas composition, and contaminants [155,156,157,158,159]. Techniques for measuring temperature, humidity, and gas composition are well developed; however, the detection of environmental contaminants, such as hydrocarbons, heavy metals, bacteria, and organic toxins is still being developed [155,156,160,161]. Sensing environmental contaminants poses numerous challenges, one consideration is that environmental contaminants are often in complex matrices, such as water or soil; therefore, many interferant components are likely present. Many matrix components may have similar chemical and physical properties to the analyte of interest. To overcome any potential matrix effects, the sensor must be highly selective [156]. Environmental contaminants and pollutants may be toxic at very low concentrations, so high sensitivity is required [156]. Another consideration for environmental sensors is that the analyst may be in the field with little laboratory experience and limited access to reagents. Given this, the sensor must be designed to be easy to use, the data must be easy to interpret, require minimal reagents to carry out analysis, and affordable [159]. Current environmental hydrogel sensor work is focused on using the physical and chemical properties of chemically modified hydrogels to detect and quantify toxic metal ions and organic compounds in environmental matrices [106,157,160,161]. Hydrogels also enable immobilization of the analytes and due to their three-dimensional cross-linked structure, they provide the possibility of incorporating highly sensitive substrates for detection [155].

### Detection of Heavy Metals

Hydrogel sensors have been used in the detection and quantification of water-soluble toxic metal ions including mercury (Hg^2+^), uranyl (UO_2_^2−^), and lead (Pb^2+^) [160,161,162,163,164]. Aqueous metal ions are of specific importance as they are very bioavailable, therefore very low concentrations can be toxic to humans and organisms in the environment [160,161,165,166].

**Figure 14:**
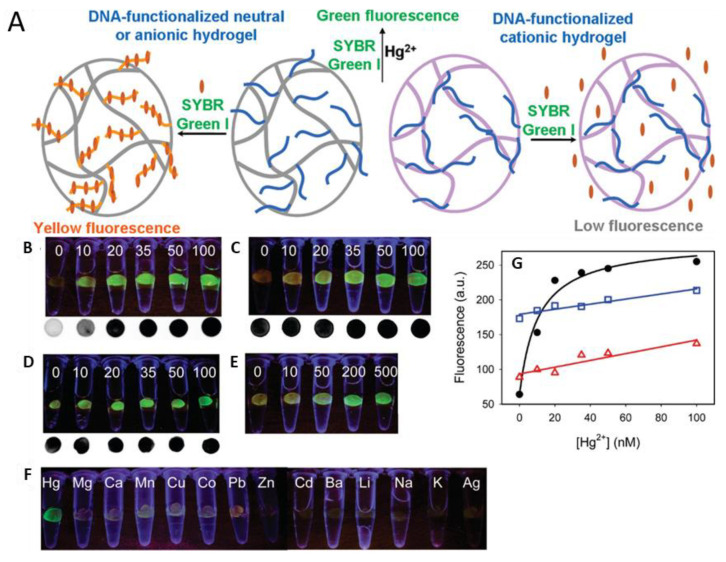
(**A**) Schematic representation of the DNA-functionalized neutral, anionic, and cationic hydrogels upon addition of fluorescence marker SYBR green. The neutral or anionic gel shows yellow fluorescence, and the cationic gel shows low fluorescence in the absence of Hg^2+^. When Hg^2+^ is present, green fluorescence can be observed. (**B**–**F**) Hydrogel mercury sensor sensitivity obtained using a digital camera (the top panels) and a gel documentation system (the lower panels) for cationic (20% allylamine) (**B**), neutral (**C**), and anionic gels (**D**). (**E**) Detection of Hg^2+^ in Lake Ontario water using the 20% allylamine gels. The numbers on the top of each sample are Hg^2+^ concentrations in nM. (**F**) Responses of the sensors quantified using the gel documentation system. Cationic gel: black dots, neutral gel, an red triangles; negative gels: blue squares. (**G**) Sensor selectivity test with 1 μM metal ion each (Reprinted and adapted with permission from [167]. Copyright 2011 American Chemical Society).

Dave et al. developed a DNA-functionalized acrylamide hydrogel sensor for the selective detection of Hg^2+^ in lake water [162]. Their sensor used the selective binding of Hg^2+^ by thiamine DNA bases followed by the addition of a fluorescent dye which turned green in the presence of Hg^2+^ and yellow in its absence. Sensing was performed using an excitation light in the ultraviolet spectrum followed by visual observation to determine if the sensor was green or yellow by using a camera or a fluorescence detector to obtain a quantitative result. The sensor can also be regenerated using a 1% HCl solution then water and buffer washes to restore the DNA hydrogel to its initial state. This sensor can also be dried to reduce its mass to about 4% of the fully hydrated gel mass. The sensor had an LOD of 75 nM Hg^2+^ using 1 mL of Hg^2+^-spiked water with a fluorescence detector. A concentration of 200 nM Hg^2+^ in 1 mL of water was required to generate a change in fluorescence which was observable to the naked eye. The authors hypothesized that acrylamide actively absorbed Hg^2+^ from the solution, therefore the sensitivity should improve upon increasing the sample volume. The sample volume was increased from 1 mL to 50 mL and the LOD was decreased to 10 nM Hg^2+^, which was easily visible. A linear response was observed from 10 to 100 nM Hg^2+^. This sensor also exhibited high selectivity. When the sensor was exposed to several aqueous heavy metals, none of them produced the green fluorescence indicative of Hg^2+^. Further work on the Hg^2+^ hydrogel sensors created by Dave et al. was carried out by Joseph et al. who optimized the original sensor by adding 20% positively charged allylamine monomer to the prepolymer mixture (Figure 14) [167]. This reduced the background fluorescence in the finished hydrogel which improved the sensitivity of the sensor [162,167]. The authors suggest the positively charged hydrogel repulsed the fluorescent dye, preventing background fluorescence when Hg^2+^ was absent, lowering the LOD to 1.1 nM Hg^2+^ [167]. Selectivity was not impacted by modifying the hydrogels’ constituency.

Qin et al. developed a hydrogel-based sensor for Hg^2+^ in sea water using polymerized colloidal photonic crystals (PCPhCs) [161]. Polystyrene colloidal nanoparticles were synthesized, characterized, then equilibrated in sea water allowing the PCPhCs to swell. The hydrogel is rich in thiol groups, which bond with Hg^2+^ and form S-Hg-S bridges, causing the PCPhCs to decrease in volume, which leads to a wavelength shift proportional to the amount of Hg^2+^. This wavelength shift was measured by collecting a diffraction spectrum before and after exposure to Hg^2+^. An unexpected behaviour in pure water was observed at low concentrations of Hg^2+^ (<500 nM). A red shift occurs, suggesting a size increase, while higher concentrations lead to a size decrease indicated by a blue shift. Therefore, in pure water there is no linear relationship between wavelength shift and Hg^2+^ concentration. The authors hypothesized that the lack of additional cations, which are present in seawater, causes a buildup of charges within the hydrogel when there is no sufficient concentration of Hg^2+^ to form S-Hg-S bridges, leading to repulsive forces within the hydrogel which would otherwise be countered by other cationic species.

In sea water, the LOD was 1 nM, which is comparable to the sensors developed by Dave et al. and Joseph et al. with a linear range of 1 nM to 100 nM [161,162,167]. The effect of different cationic species in water spiked with Hg^2+^ was studied to see how interferences affected the blue shift. This revealed that different metal ions affected the blue shift of the sensor, which was attributed to hard–soft acid-base theory [161]. This presents a selectivity issue as the matrix composition will affect the response; for instance, if sampling occurs in a Cd^2+^-containing body of water, the blue shift will increase, therefore leading to a higher concentration of Hg^2+^ being measured than is actually present.

As visual detection is a desirable feature of sensors, a “smart switch sensor” hydrogel (SWSH) was prepared by incorporating nitrogen-doped carbon quantum dots (N-CQDs) into a polyacrylamide (PAM) hydrogel network with functionalities of easy handling, recyclability, and simultaneously recognizing dual metal ions. The prepared SWSH showed a reversible fluorescence “off/on” response with Cu^2+^/Ag^+^ and L-cysteine (L-cys), and the limit of detection values of the SWSH for these metal ions were 0.813, 0.468, and 0.437 µM, respectively [168]. In another approach, a chemosensor has been developed based on a safranin-impregnated PVA hydrogel for the visual recognition of copper ions (Cu^2+^). Here, the Cu^2+^ recognition is based on a colour change of safranin from basic red to yellow, with the blue shift in the absorption peak owing to the formation of a complex between Cu^2+^ and safranin. The selectivity response of safranin was tested towards different metal ions and it exhibited elevated selectivity for Cu^2+^ among the tested metal ions. The LOD of the chemosensor probe was calculated and established to be 8.6 ± 0.004 µM [169].

Uranium is a hazardous heavy metal due to both its chemical properties and its radioactivity [163]. Uranyl (UO_2_^2+^) is a water-soluble uranium oxide found in the environment that is toxic at low concentrations [163,170]. He et al. developed a hydrogel-based SERS sensor for the determination of uranyl [163]. The Raman cross-section of uranyl is small which leads to a lower Raman signal, therefore He et al. combined DNA hydrogels with SERS to enhance the signal. In the DNA hydrogel, the DNA is grafted to the polyacrylamide chain with a DNAzyme substrate sequence prolonged at both ends. The hydrogel was cross-linked by both substrate and enzyme strands with the polyacrylamide hydrogel making up the scaffolding. Raman reporter rhodamine B (RhB) was trapped in the hydrogel which rendered it inactive. The hydrogel was placed on a polyacrylonitrile (PAN) array which can capture free RhB. The introduction of uranyl ions triggered the cleavage of the substrate strand from the enzyme strand releasing the RhB, as the cleavage destabilizes the hydrogel structure. The release of RhB was then detected using Raman spectroscopy. The LOD of the detector is 0.838 pM of uranyl ions. This is quite sensitive compared to other DNA hydrogel metal ion sensors [162,163,166,171]. The selectivity was determined by exposing the sensor to 15 interfering metal ions at high concentrations (10 and 100 μM). Very weak Raman intensities were observed for all these metal ions, demonstrating the selectivity of this technique [163]. Fish and kelp samples were exposed to uranyl ions at nano-molar levels then recovered and analyzed. These spiked samples all produced a sufficient signal for the quantification of uranyl; however, the authors did not perform quantification to determine the percent recovery. This uranyl sensor also has the potential to be used in the field for monitoring uranyl contamination as Raman instruments have become more portable in recent years [163,172].

Xiao et al. designed a smart photonic crystal hydrogel (PhCH) for uranyl ion detection [173]. This PhCH was synthesized by dispersing 160 nm silica microspheres in a custom hydrogel which contained acrylamide, poly(ethylene glycol), and *N,N′*-methylenebisacrylamide, providing a number of amide sites for the binding of uranyl ions. This PhCH was hydrolyzed to add carboxyl groups to the hydrogel network which increased the shrinkage ratio of the hydrogel when exposed to uranyl. UV-Vis spectroscopy was used to determine the diffraction shift resulting from shrinkage of the sensor when exposed to uranyl and other interferent ions. The LOD for this sensor was 10 nM, which is orders of magnitude higher than the sensor developed by He et al. [163]. The linear range was wide, covering three orders of magnitude from 10 nM to 300 μM [173]. Selectivity was sufficient when the sensor was exposed to numerous interferent ions, with the maximum diffraction shift from an interferent being equivalent to 40 nM of uranyl. Much like the hydrogel sensor described in the work of Dave et al., this sensor can be regenerated by exposure to an aqueous acid, while He et al. did not test whether their sensor can be regenerated or not [162,163,173]. It is unlikely that He’s sensor can be regenerated since RhB was introduced to the hydrogel during the synthesis [163]. While this uranyl sensor is much less sensitive than the sensor designed by He et al., the diffraction shift of the sensor synthesized by Xiao et al. results in a colour change which can be observed without instrumentation, similar to the mercury sensor prepared by Dave et al. [162,163,173].

Lead (Pb^2+^) pollution in the environment is important due to the many known health hazards it poses, including damage to the central nervous system [174]. The upper limit of lead in water is 72.4 nM, therefore a selective lead sensor needs to be sensitive enough to reliably quantify Pb^2+^ at this concentration [175]. Huang et al. developed a DNAzyme cross-linked hydrogel sensor for the detection of Pb^2+^, similar to the uranyl sensor developed by He et al. [163,175]. Huang et al., took advantage of a Pb^2+^-dependent DNAzyme, which is highly selective for Pb^2+^ as it is its cofactor [175]. When the DNAzyme is exposed to Pb^2+^ it becomes activated and cleaves the substrate sequence which destabilizes the hydrogel, releasing gold nanoparticles which are indicators for colourimetric detection. These released AuNPs also efficiently catalyze the degradation of H_2_O_2_ to generate O_2_. The generation of O_2_ is used in a volumetric bar-chart chip (V-chip) for quantitative visual detection; the more O_2_ that is produced, the further the ink up the V-chip [175,176]. The LOD for UV-Vis spectroscopy was 3.4 nM and 5 nM for visual detection using the V-chip. Selectivity experiments using eight common interferent cations at 1 mM found no absorbance or visual changes in the sensor demonstrating the highly selective nature of this sensor [175]. Additionally, in sea water, which is a relatively complex matrix, Pb^2+^ concentrations as low as 30 nM were detected, demonstrating the ability for this sensor to be used for the analysis of real samples.

Chu et al. developed a pure DNA hydrogel for the detection of Pb^2+^ without the need for signal-tags such as the AuNPs used by Huang et al. [175,177]. A lead dependent DNAzyme strand and a substrate strand were incorporated into a pure DNA hydrogel [177]. When Pb^2+^ is introduced to the sensor, the DNAzyme catalyzes the degradation of the hydrogel which released oligonucleotide fragments. These released oligonucleotide fragments can be detected by UV-Vis spectroscopy. An LOD of 7.7 nM was established with a linear range of 0–500 nM. The addition of 8 interferent cations at 200 and 500 nM resulted in the release of very few DNA fragments, with a signal comparable to the blank sample. Accuracy was assessed by spiking tap water with 100, 200, and 500 nM Pb^2+^ which resulted in 107%, 113%, and 90% recovery, respectively. Pb^2+^-dependent DNAzyme was utilized in the work of Jiang et al. to change the behaviour of sample flow in a tube [166]. The DNA hydrogel is at the end of the capillary tube preventing the flow of liquid. When the hydrogel is exposed to Pb^2+^, the DNAzyme cleaves the substrate leading to the flow of solution through the hydrogel. The distance of flow within the capillary tube is proportional to the concentration of Pb^2+^ in the solution, which can be noted visually or using image processing software. The LOD was established as 10 nM with a linear range of 10 nM to 50 µM. Exposure of the sensor to various metal cations had a negligible effect on the sensor. Lead detection using hydrogel-based sensors is commonly performed using DNA-based hydrogels, as certain DNAzymes and oligomers have excellent selectivity for Pb^2+^ binding [166,175,177]. Another DNA-based hydrogel sensor for Pb^2+^ by Jacobi et al. also took advantage of this selective binding of Pb^2+^ and the fluorescence shift it induced to DNA after being exposed to several different dyes (e.g., SYBR Green I PicoGreen, SYTO-13, ethidium bromide, Hoechst and thiazole orange) [178]. These four hydrogel sensors had comparable LODs and selectivity due to all these sensors utilizing the ability of DNA to bind Pb^2+^. Additionally, all of these sensors allow for the visual detection of Pb^2+^, with the sensors by Jiang et al. and Chu et al. allowing for visual quantification down to nM concentrations [166,175,177,178]. All of these Pb^2+^ sensors are easy to customize in order to use in the field, requiring little user experience and additional reagents, with the user obtaining results within 2 h.

Concern about the release of many different organic pollutants into freshwater or the environment, such as in agriculture, is growing. Given this, there is an increasing need for big assay sensing methods to detect even the smallest amounts of pharmaceuticals or pesticides and other organic compounds in freshwater or agricultural products [121,122,179]. There is also an increasing interest in sensing applications for gas because conventional methods do not work properly under humid conditions yet or have a great consumption of resources, such as the use of complex equipment, time, and professionals [158,180]. Another approach is the removal of the toxic substances even though this could be realized with hydrogels [181].

**Figure 15:**
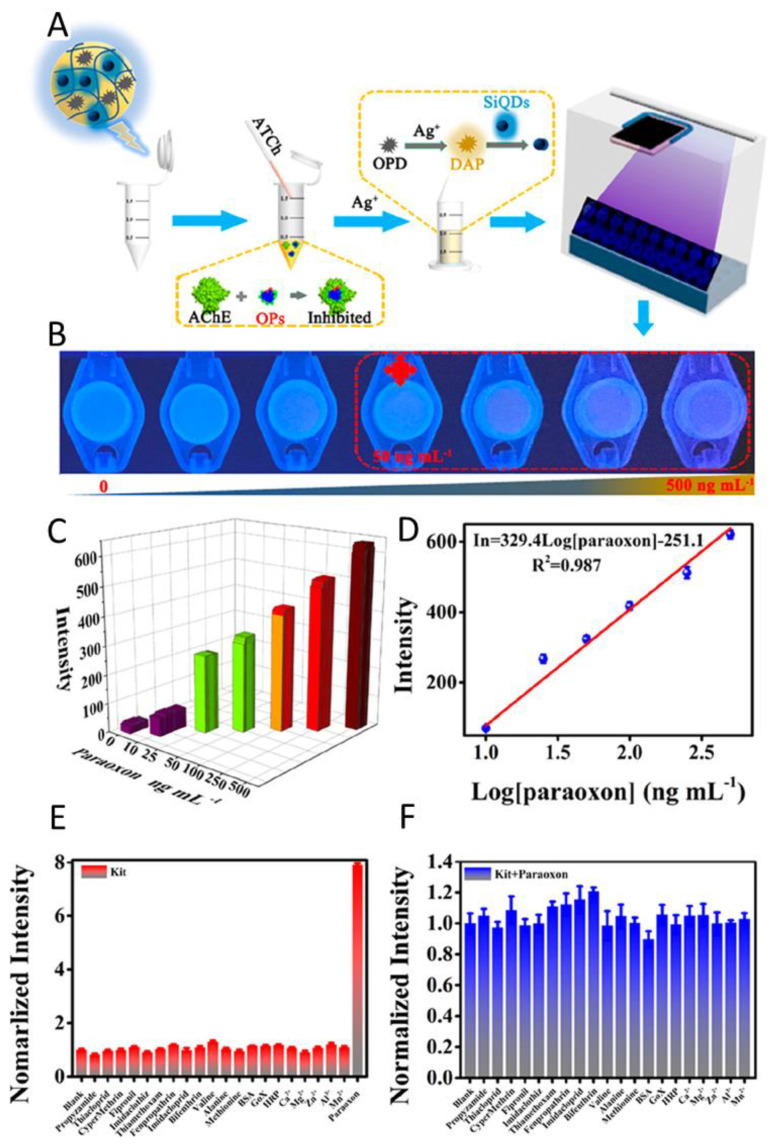
(**A**) Program for detecting OPs. (**B**) Optical images of test kits. (**C**) Original hue intensity of kit digitally analyzed by ImageJ software. (**D**) Relationship between hue intensity and the logarithm of concentration of paraoxon (10, 25, 50, 100, 250, and 50 ng/mL). (**E**) Portable kit in the presence of interfering substances. (**F**) Portable kit for pesticide detection with the coexisting substances (Reprinted with permission from [182]. Copyright 2019 American Chemical Society).

Pesticides are widely used to prevent weed growth or pest infestation in agriculture. Organophosphorus pesticides (OPs) have a bad impact on human health by inhibiting the enzyme acetylcholinesterase (AChE). To have novel methods to detect these pesticides levels in food is a desired goal. Jin et al. provided a hydrogel which will emit fluorescent light when an OP is incorporated [182]. The portable tool works with an Ag^+^-sensitive hydrogel, consisting of o-phenylenediamine with silicon quantum dots as a fluorescent indicator. Both were carried by an agarose gel in the snap caps of Eppendorf tubes (Figure 15). The linear detection range reached from 10 to 500 ng/mL with an LOD of 10 ng/mL. There are comparable results with a gold nanorod-based paper device [183]. However, with the response time of 45 min, the hydrogel approach is about 30% faster [182]. In the sensitive system, Ag^+^ is released when the OP is inhibiting the AChE. The silver ions diffused into the hydrogel and oxidized the o-phenylenediamine to 2,3-diaminophenazine (DAP). The DAP was excited in UV at 365 nm and fluorescent light was strongly emitted at 557 nm.

Another promising way of using hydrogels for OP detection is provided by Lu et al. [179]. They produced a hydrogel-based chemiluminescence sensor to detect pesticides and D-amino acids (D-AAs). Both OP and D-AAs are harmful to human health; D-AAs seem to be relevant in Alzheimer’s diseases, epilepsy, and schizophrenia. AChE and choline oxidase react with acetylcholine chloride, releasing H_2_O_2_ in the process. This caused luminescence when reacted with *N*-(4-aminobutyl)-*N*-ethyl isoluminol/Co^2+^/chitosan. When an OP is present, the AChE is inhibited, and the luminescence will have a lower intensity. By adding a metal organic framework (MOF) based on Pt, the signal increased six times due to the catalytic activity of Pt. The detection of D-AAs works the opposite way, by increasing H_2_O_2_ production during their oxidase reaction. This will lead to an increased chemiluminescence intensity. For chlorpyrifos, they could achieve a LOD of 0.21 ng/mL and a linear quantification range from 0.5 ng/mL to 1.0 µg/mL, and they achieved detection of 17 kinds of OPs. The D-AAs, using D-Alanine as a model analyte, established an LOD of 0.12 µM and a linear range of 1.0 µM to 10 mM. Future work will include the observation of response factors of different OPs and D-AAs. The LOD in the chemiluminescence sensor of Lu et al. was about two orders of magnitude lower than that of Jin et al., with a linear range nearly twice as wide [179,182]. Additionally, Lu et al. simultaneously developed a detection method for D-AAs as well [179].

The capability of hydrogels to withstand humid conditions is desired for realistic gas sensing applications [158]. Gases could exist in humidified form which normally is an issue for the sensor itself. If a hydrogel is becoming attached to a silicon surface it can inhibit the degradation of the sensitive surface. Yu et al. addressed an application like this by using the hydrogel as a humidity regulator [158]. The group proposes that having a water-rich hydrogel could prevent the noise signals or humidity variations in colourimetric detectors for gas sensing. They could show that the hydrogel-incorporated sensor was able to stabilize the signal from the detection device sensing for carbon monoxide. Without the hydrogel, there was no possibility for a linear correlation. Even the durability of the sensors could be maintained over a longer storage time than the uncoated ones. Another advantage is that the detector becomes independent from the flow rate because the hydrogel transfers the detection reaction from a gas–solid phase to a gas–liquid phase reaction.

Hong et al. presented another approach with a hydrogel to detect CO_2_ [180]. The hydrogel formed an inverse opal which shrinks or swells depending on the conditions; thus, a wavelength shift could be observed. The synthesis of the dimethyl aminopropyl methacrylamide hydrogel was accomplished by photopolymerization in the crystalline voids of a silica opal (diameter: 190 nm, reflection maximum: 448 nm) on a glass slide. The opal itself was removed with 1% hydrofluoric acid and the inverse hydrogel opal was obtained. Before, there were problems with the detection of CO_2_ under humid conditions, but in the hydrogel, there is no more interference from analytes such as CO. Furthermore, they are able to reliably detect CO_2_ in the presence of basic or acidic gases (<500 ppm), which usually would cause a swelling of the hydrogel as well. The hydrogel even performed under realistic measurements of exhaust gasses from a diesel engine and the results could fit in the obtained linear range. When acidic fouling is exposed to the hydrogel film it is possible to refresh it with a solution of pH 11. The temperature interference as well as the lifetime (3 months) do not seem to have any significant impact on the sensor’s accuracy. The LOD was measured with 0.2 vol% for a 1 mL gas sample, with the authors suggesting that increasing the volume will obtain a higher LOD.

## 5. Conclusions

Numerous novel sensors incorporating hydrogels into their design have been developed recently to detect biomarkers of disease, DNA, pharmaceuticals, and environmental contaminants. The sensors explored in this review require little operator training, additional reagents, and complex instrumentation to achieve accurate and sensitive qualification and quantification of the desired analytes in the field. As the majority of these sensors rely on colourimetric detection, detection can be performed visually or with the aid of a smart phone camera. The incorporation of selective DNA oligomers, ligands, quantum/carbon dots, or aptamers allows for this selective and sensitive detection and quantification of targeted analytes. Research in this field is evolving, especially with respect to the detection of organic compounds in complex matrices; therefore, we expect future research to explore techniques to improve detector selectivity and robustness in a variety of matrices while preserving sensor sensitivity.

## Data Availability

Not applicable.
